# Supplementary material for: Accurate and efficient estimation of local heritability using summary statistics and the linkage disequilibrium matrix
Source: Nat Commun. 2023 Dec 2;14:7954. doi: 10.1038/s41467-023-43565-9 (PMC10692177; doi:10.1038/s41467-023-43565-9)
Supplement: Supplementary file 1 — Supplementary Information [file 41467_2023_43565_MOESM1_ESM.pdf]

Supplementary Notes

Hui Li[1], **Rahul Mazumder**[2], **and Xihong Lin**[1,3]

[1]Harvard T.H. Chan School of Public Health, Department of Biostatistics, Boston, USA
[2]Massachusetts Institute of Technology, Operations Research and Statistics group, Cambridge, USA
[3]Harvard University, Department of Statistics, Cambridge, USA
Corresponding author: xlin@hsph.harvard.edu

Supplementary Note 1

Here we provide a more comprehensive comparison of the existing heritability estimation methods (summarized in Supplementary Table 1). It is not our intention to compare HEELS with all or most of the existing heritability estimation methods, but we hope to highlight the similarities and differences among approaches in terms of statistical efficiency.

Existing heritability estimation methods using individual-level data

Linear mixed models (LMMs) has been widely used as the analytical framework for h^2_{SNP} estimation. In a nutshell, h^2_{SNP} is embedded in the parameters of a LMM as the variance of causal genetic effects. One of the most commonly used variance component estimation methods is restricted maximum likelihood (REML). The well-known tool of Genome-wide Complex Traits Analysis (GCTA) applies this method in the context of genetics studies, which led to the "GREML" estimator of SNP-heritability[1]. GREML uses second-order methods (average information or Fisher scoring) to maximize the log-likelihood function based on individual genotypic and phenotypic data. The solutions are found through iterative optimization algorithms, which can be computationally intensive when applied to large samples.

To overcome the computational limitations GREML, Loh *et al.* introduced BOLT-REML, which is a highly efficient algorithm that enables robust and fast variance component estimation on biobank-scale datasets[2]. BOLT-REML greatly improves the computational efficiency of the estimation procedure of GREML, by approximating the gradient (and the Hessian matrix) of the likelihood function via Monte Carlo sampling. It also adopts numerical techniques such as the conjugate gradient algorithm for mixed-model solutions to reduce its computational cost[2].

Speed *et al.* made an important advance in heritability estimation, by introducing a new REML estimator of h^2_{SNP}, called LD-adjusted kinships (LDAK)[3]. LDAK differs from GREML in its assumption of the relationships between the effect sizes of causal markers and their minor allele frequencies (MAF) as well as LD tagging. The main advantage of REML estimators, such as GREML and LDAK, is that they have high statistical efficiency – because they produce MLE or REML estimators, especially in large samples, the asymptotic variance of these two estimators is guaranteed to attain the Cramer-Rao lower bound. Previous works have shown that the stratified or multi-component GREML can effectively account

for the coupling relationship between causal effect sizes and MAF/LD structure[4]. Therefore, the GREML estimator can be reconciled with the LDAK estimator under certain assumptions[5].

Haseman–Elston (HE) regression is another well-known approach for estimating heritability[6,7], which is germane to the method-of-moments (MoM) estimators of h^2_{SNP}, *i.e.*, population covariance of the phenotypes is matched to the empirical covariance[8]. The computational bottleneck of applying the HE-regression is its requirement to calculate a large genetic relatedness matrix that summarizes the relationship between all $n \times n$ pairs of individuals in the sample, especially when sample size exceeds 30,000[1]. Wu et al. developed a randomized HE-regression (RHE-reg) estimator that reduces both the runtime and the storage requirement of heritability estimation based on HE-regression[9]. The main contribution of their approach pertains to the usage of random sampling for trace calculation and its application of the Mailman algorithm for matrix-vector multiplication to solve the MoM normal equations. Both BOLT-REML and RHE-reg employ random sampling to circumvent frequent and expensive operations that involve high-dimensional matrices. The downside of such approximation is that they tend to produce less statistically efficient estimators, due to the additional variance introduced by randomization[2,9].

Another important line of research estimates SNP-heritability under the Bayesian framework, which adopts different assumptions about the distribution of true causal effect sizes. The first method of this kind is the sparse regression model proposed by Guan and Stephens[10]. They introduced "Bayesian variable selection regression (BVSR)" for SNP-heritability estimation and phenotypic prediction. BVSR assumes that the joint effect size follows a point-normal distribution, and it works well when the genetic architecture of a trait is truly sparse. Later, Bayesian Sparse Linear Mixed Model (BSLMM) was proposed as a hybrid approach that combines the advantages of LMM and BVSR[11]. BSLMM uses a mixture of two normals to jointly model a small number of large effects ("sparse" component) and a large number of small effects ("polygenic" component). A key advantage of the Bayesian framework is its flexibility to model different effect size distributions. For example, the Bayesian alphabet[12] models is a class of variants to BSLMM and BSVR which make different distributional assumptions on the effect sizes, with the BayesCπ being the closest to BSLMM[13]. The main challenge of using these Bayesian methods however, is that the posterior inference procedure can be quite computationally intensive, *e.g.*, orders of magnitude slower than LMM when applied to large-scale genetic data[11].

Existing heritability estimation methods using summary statistics

When only GWAS summary-level statistics are available, LD score regression (LDSC) is a state-of-the-art method that is highly computationally efficient and yields heritability estimates that can adjust for confounding by environmental effects or population stratification[14,15]. From the modeling perspective, LDSC is similar to GREML as it also adopts the LMM and assumes that all causal markers contribute to heritability equally. From the estimation perspective, the LDSC estimator of heritability is akin to RHE-reg, as both methods model the second moments of the effect size without making any distributional assumptions. The most important contribution of LDSC is its ability to distinguish confounding bias

from true polygenic genetic signals, although a recent study raised some concern about this capacity[16]. SumHer[17] was introduced as a summary-statistics-based extension of LDAK, as it explicitly accounts for the MAF/LD-dependent structure of effect sizes. The comparison between LDSC and SumHer is parallel to the comparison between GREML and LDAK, and recent works have shown the converging performance of these two lines of research with the usage of stratification[4,5,18].

Although summary-statistics-based methods are attractive due to its broader applicability and lessened privacy concern, an important drawback of these approaches is that they generally produce estimates with considerably large standard errors than individual-level data base REML estimates. For example, Zhou *et al.* demonstrated within the framework of Minimal Norm Quadratic Unbiased Estimation (MINQUE) that the weight matrix used by LDSC in solving the MoM normal equations is not optimal, and therefore its estimator does not achieve the highest statistical efficiency[19]. We show the statistical efficiency of our HEELS by establishing its equivalence with the generalized methods-of-moment estimator with the optimal weights under the MINQUE framework. A new MoM estimator – MinQue for Summary Statistics (MQS) – was introduced under this unified framework[19]. The paper shows that MQS can improve the statistical efficiency of LDSC under certain settings, which is affected by the weighting scheme, true heritability as well as the degree of relatedness in the sample[19]. MQS is unbiased and is generally more statistically efficient than LDSC, but still has larger variance than REML (see Figure 2 and Table 2 of[19]).

Two other h^2_{SNP} estimators resemble HEELS and are based on summary-level statistics – Generalized Random Estimator (GRE[20]) and Heritability Estimator from Summary Statistics (HESS[21]). Both of these two methods produce closed-form solutions of h^2_{SNP}, and are robust to the unknown underlying genetic architecture of the phenotype. Although HESS was developed using a fixed-effect model whereas GRE assumes the causal effect sizes are random, the derivation of these two estimators are closely related, as is evident from their analytical expressions (see later sections). When applied to a local genetic region, h^2_{HESS} coincides with h^2_{GRE} when the in-sample LD is used without any regularization (*i.e.* the number of top eigenvectors used by HESS equals to the rank of the LD matrix).

The summary-statistics-based extension of the Bayesian methods such as BVSR and BSLMM is Regression with Summary Statistics (RSS)[22]. RSS adopts similar assumptions as BSLMM but uses the likelihood of the joint effect sizes and performs inference on the SNP-heritability defined using summary statistics. RSS also leverages the banded structure of the LD and uses the shrinkage estimator from Wen and Stephens[23] to approximate LD. The simulation results suggest that RSS is unbiased but is less statistically efficient than its corresponding individual-level data based method (see Figure 3 of[24]).

A recently developed method, High-Definition Likelihood ("HDL"), shares our goal of improving the statistical efficiency of genetic variance estimators[25]. Although HDL was proposed as a method for genetic correlation estimation, it necessarily computes heritability as an intermediate step. In simulations, we observed bias in HDL's estimates of heritability, and another study has reported similar issues in their benchmarking results[26]. Our approach differs from HDL in two important ways. First, our estimator was derived using a different likelihood function (*i.e.*, we start with the likelihood that assumes individual-level

data is known, and then transform the score-solving algorithm into a summary-statistics-based estimating procedure). HDL first derived the likelihood of the marginal statistics, which is closely related to the "RSS" likelihood proposed in Zhu and Stephens[24]. Second, HDL approximates the LD matrix using a combination of banding, blocking and truncated SVD, whereas we proposed approximating the LD matrix in a principled manner using a banded + low-rank representation.

We summarize the SNP-heritability estimator methods reviewed above in Supplementary Table 1. We select GREML, LDSC, GRE and HESS as the representative heritability estimation methods to be compared with our HEELS, because the differences between these methods and the other existing approaches lie elsewhere from *statistical efficiency*. For methods that we do not directly compare with HEELS, we cite existing evidence of the statistical efficiency (see the last column of Supplementary Table 1). We do not include HDL in our comparisons of summary-statistics-based estimators due to the bias we observed and reported elsewhere, as mentioned above.

Supplementary Note 2

Here, we describe the model and the REML likelihood, assuming that the individual-level data is available. Let $\mathbf{y}$ be a length-n vector that denotes the phenotypes of n samples. Denote by $\mathbf{X} \in \mathbb{R}^{n \times p}$ the genotype matrix of n individuals based on p markers or SNPs. We standardize $\mathbf{X}$ and $\mathbf{y}$ such that the variance of the phenotype is 1 and the variance of each marker-specific genotype vector is $1/p$, or $diag(\mathbf{X}^\top \mathbf{X}/n) = 1/p$. Let $\mathbf{S}$ and $\mathbf{R}$ denote the the marginal association statistics and the in-sample LD matrix, *i.e.* $\mathbf{S} = \mathbf{X}^\top \mathbf{y}$ and $\mathbf{R} = \mathbf{X}^\top \mathbf{X}$. (Note that our definitions here omit the scaling by $1/\sqrt{n}$ for $\mathbf{S}$ or $1/n$ for $\mathbf{R}$. Because we assume an *in-sample* LD, the scaling does not affect the derivation of the HEELS estimator. Hence we define the statistics **S,R** without involving n for simplicity of exposition.) Our goal is to develop a heritability estimator using the two statistics $(\mathbf{S}, \mathbf{R})$, which attains comparable statistical efficiency as the REML estimator based on individual-level data $(\mathbf{X}, \mathbf{y})$. We start by considering the likelihood function, assuming individual-level data can be accessed.

We use an additive genetic model for the phenotypes as $\mathbf{y} = \mathbf{X}\beta + \varepsilon$, where is a $p \times 1$ vector assumed to follow $N(0, \sigma_g^2 \mathbf{I}_p)$, and ε is a length-n vector distributed as $\varepsilon \sim N(0, \sigma_e^2 \mathbf{I}_n)$. Under these assumptions, $\mathbf{y} \sim N(0, \mathbf{V})$, where the variance-covariance matrix is $\mathbf{V} \equiv var(\mathbf{y}) = \sigma_g^2 \mathbf{X}\mathbf{X}^\top + \sigma_e^2 \mathbf{I}_n$. We define SNP-heritability conditional on $\mathbf{X}$ as the following,

$$h^2_{SNP} := \frac{Var(\mathbf{X}|\mathbf{X})}{Var(\mathbf{y}|\mathbf{X})} = \frac{Var(\mathbf{X}|\mathbf{X})}{Var(\mathbf{X}|\mathbf{X}) + \sigma_e^2} = \frac{tr(\sigma_g^2 \mathbf{I_p} \mathbf{X}^\top \mathbf{X})/n}{tr(\sigma_g^2 \mathbf{I_p} \mathbf{X}^\top \mathbf{X})/n + \sigma_e^2} = \frac{\sigma_g^2}{\sigma_g^2 + \sigma_e^2}.$$

The log-likelihood function for (σ_g^2, σ_e^2) is,

$$\ell(\mathbf{y}; \sigma_g^2, \sigma_e^2) = -\frac{1}{2} ln|\mathbf{V}| - \frac{1}{2} \mathbf{y}^\top \mathbf{V}^{-1} \mathbf{y}. \qquad (1)$$

Using well-known results in matrix differentiation, we can maximize this log-likelihood with respect to σ_g^2 and σ_e^2, by solving the following score equations[27],

$$U_{\sigma_g^2}(\mathbf{y}) = -\frac{1}{2}tr(\mathbf{X}^\top\mathbf{V}^{-1}\mathbf{X}) + \frac{1}{2}\mathbf{y}^\top\mathbf{V}^{-\mathbf{1}}\mathbf{X}\mathbf{X}^\top\mathbf{V}^{-\mathbf{1}}\mathbf{y} = \mathbf{0} \tag{2}$$

$$U_{\sigma_e^2}(\mathbf{y}) = -\frac{1}{2}tr(\mathbf{V}^{-1}) + \frac{1}{2}\mathbf{y}^\top\mathbf{V}^{-\mathbf{1}}\mathbf{V}^{-\mathbf{1}}\mathbf{y} = \mathbf{0} \tag{3}$$

Henderson developed a set of equations[28,29], known as the mixed model equations (MME), which maximize the joint density of the outcomes and the random effects,

$$\ell(\mathbf{y},\beta;\sigma_g^2,\sigma_e^2) = -\frac{1}{2\sigma_e^2}(\mathbf{y}-\mathbf{X}\boldsymbol{\beta})^\top(\mathbf{y}-\mathbf{X}\boldsymbol{\beta}) - \frac{1}{2\sigma_g^2}\boldsymbol{\beta}^\top\boldsymbol{\beta} - \frac{n}{2}log(\sigma_e^2) - \frac{p}{2}log(\sigma_g^2). \tag{4}$$

The Best Linear Unbiased Predictor (BLUP), which are estimates for the random effects from these MMEs, can be plugged into the Supplementary Equation (2)-(3) to generate an iterative procedure for estimating the variance components[27,30]. We exploit the "dual" form of this algorithm, which gives rise to the HEELS estimator. Note that for simplicity, we have assumed all of the observable environmental factors have been projected out, but covariates (*i.e.* fixed effects) can be easily incorporated into the model by adopting the restricted maximum likelihood approach and using the projection matrix[31,32].

Supplementary Note 3

The marginal likelihood in the Supplementary Equation (1) can be expressed using the joint likelihood in Supplementary Equation (4) and the probability of the causal effects, using the partition theorem,

$$\begin{aligned} f(\mathbf{y}) &= \int f(\mathbf{y},)d = \int f(\mathbf{y}|)f()d \\ &= \int (2\pi\sigma_g^2)^{-p/2}(2\pi\sigma_e^2)^{-n/2}exp\left(-\frac{1}{2}\left(\frac{1}{\sigma_e^2}(\mathbf{y}-\mathbf{X})^\top(\mathbf{y}-\mathbf{X}) + \frac{1}{\sigma_g^2}{}^\top\right)\right)d \\ &= \left|\mathbf{X}^\top\mathbf{X} + \frac{\sigma_e^2}{\sigma_g^2}\mathbf{I}\right|^{p/2}(2\pi\sigma_e^2/\sigma_g^2)^{p/2}(2\pi\sigma_e^2)^{-n/2}exp\left(-\frac{1}{2\sigma_e^2}\left(\mathbf{y}^\top\mathbf{y} - \mathbf{y}^\top\mathbf{X}\left(\mathbf{X}^\top\mathbf{X} + \frac{\sigma_e^2}{\sigma_g^2}\mathbf{I}\right)^{-1}\mathbf{X}^\top\mathbf{y}\right)\right). \\ &\qquad \text{(use the kernel of } \sim N\left(\left(\mathbf{X}^\top\mathbf{X} + \tfrac{\sigma_e^2}{\sigma_g^2}\mathbf{I}\right)^{-1}\mathbf{X}^\top\mathbf{y}, \tfrac{1}{\sigma_e^2}\mathbf{X}^\top\mathbf{X} + \tfrac{1}{\sigma_g^2}\mathbf{I}\right)) \end{aligned}$$

Therefore, taking the log and omitting the constant term, we can rewrite Supplementary Equation (1) as a function of summary statistics, given the unknown variance components,

$$\ell_{HEELS}(\mathbf{S},\mathbf{R};\sigma_g^2,\sigma_e^2) = -\frac{1}{2}log\left|\sigma_e^2\mathbf{I}_n\right| - \frac{1}{2}log\left|\mathbf{I}_p + \frac{\sigma_g^2}{\sigma_e^2}\mathbf{R}\right| - \frac{1}{2\sigma_e^2}\left(\mathbf{y}^\top\mathbf{y} - \mathbf{S}^\top\left(\frac{\sigma_e^2}{\sigma_g^2}\mathbf{I}_p + \mathbf{R}\right)^{-1}\mathbf{S}\right). \tag{5}$$

HEELS uses the marginal association statistics $\mathbf{S}=\mathbf{X}^{\top}\mathbf{y}$ and the LD matrix $\mathbf{R}=\mathbf{X}^{\top}\mathbf{X}$ to solve for the variance component estimates that maximize the likelihood in Supplementary Equation (5), alternating between updating the BLUP estimates $\hat{}(\widehat{\sigma_g^2},\widehat{\sigma_e^2})=\left(\frac{\widehat{\sigma_e^2}}{\widehat{\sigma_g^2}}\mathbf{I}_p+\mathbf{R}\right)^{-1}\mathbf{S}$ and updating the variance component estimates $(\widehat{\sigma_g}^2,\widehat{\sigma_e}^2)$ until convergence. Below we provide the full details for deriving the updating equations used in HEELS, for which we referenced Chapter 7 of Searle, Casella and McCulloch[33] as well as Harville (1977)[27].

Updating equation for β

The BLUP estimates that maximize the joint density in Supplementary Equation (4) satisfy $\left(\frac{\mathbf{R}}{\sigma_e^2}+\frac{1}{\sigma_g^2}\right)=\frac{1}{\sigma_e^2}\mathbf{S}$. Therefore, if the variance components are given and known, we can calculate the BLUP estimates as $=\left(\frac{\sigma_e^2}{\sigma_g^2}\mathbf{I}_p+\mathbf{R}\right)^{-1}\mathbf{S}$. This is essentially our updating equation for the joint effect sizes if we replace the parameters with their current estimates at each iteration.

Updating equation for σ_e^2

Define $\mathbf{H}=\frac{\mathbf{V}}{\sigma_e^2}$, so that $\mathbf{V}^{-1}=\frac{\mathbf{H}^{-1}}{\sigma_e^2}$. We can then establish the following:

$$\mathbf{X}\mathbf{X}^{\top}\sigma_g^2=\mathbf{V}-\sigma_e^2\mathbf{I}=(\mathbf{H}-\mathbf{I})\sigma_e^2.$$

Setting $U_{\sigma_g^2}=\mathbf{0}$, we have:

$$\begin{aligned}
tr(\mathbf{X}^{\top}\mathbf{V}^{-1}\mathbf{X}) &= (\mathbf{X}^{\top}\mathbf{V}^{-1}\mathbf{y})^{\top}(\mathbf{X}^{\top}\mathbf{V}^{-1}\mathbf{y})\\
tr(\mathbf{V}^{-1}\mathbf{X}\mathbf{X}^{\top}\sigma_g^2) &= \mathbf{y}^{\top}\mathbf{V}^{-1}\mathbf{X}\mathbf{X}^{\top}\sigma_g^2\mathbf{V}^{-1}\mathbf{y}\\
tr(\mathbf{V}^{-1}\mathbf{X}\mathbf{X}^{\top}\sigma_g^2) &= \mathbf{y}^{\top}\mathbf{V}^{-1}(\mathbf{V}-\sigma_e^2\mathbf{I})\mathbf{V}^{-1}\mathbf{y}\\
tr(\mathbf{V}^{-1}\mathbf{X}\mathbf{X}^{\top}\sigma_g^2) &= \mathbf{y}^{\top}\mathbf{V}^{-1}(\mathbf{V}-\sigma_e^2\mathbf{I})\mathbf{V}^{-1}\mathbf{y}\\
tr(\mathbf{V}^{-1}\mathbf{X}\mathbf{X}^{\top}\sigma_g^2) &= \mathbf{y}^{\top}\mathbf{V}^{-1}(\mathbf{H}-\mathbf{I})\sigma_e^2\mathbf{V}^{-1}\mathbf{y}\\
tr\left(\frac{\mathbf{H}^{-1}}{\sigma_e^2}(\mathbf{H}-\mathbf{I})\sigma_e^2\right) &= \mathbf{y}^{\top}\frac{\mathbf{H}^{-1}}{\sigma_e^2}(\mathbf{H}-\mathbf{I})\sigma_e^2\frac{\mathbf{H}^{-1}}{\sigma_e^2}\mathbf{y}\\
tr(\mathbf{H}^{-1}\mathbf{H})-tr(\mathbf{H}^{-1}) &= \frac{1}{\sigma_e^2}(\mathbf{y}^{\top}\mathbf{H}^{-1}\mathbf{y}-\mathbf{y}^{\top}\mathbf{H}^{-1}\mathbf{H}^{-1}\mathbf{y})\\
n\sigma_e^2-tr(\mathbf{H}^{-1})\sigma_e^2 &= \mathbf{y}^{\top}\mathbf{H}^{-1}\mathbf{y}-\mathbf{y}^{\top}\mathbf{H}^{-1}\mathbf{H}^{-1}\mathbf{y}\\
n\sigma_e^2 &= \mathbf{y}^{\top}\mathbf{H}^{-1}\mathbf{y}+tr(\mathbf{H}^{-1})\left(\sigma_e^2-\frac{\mathbf{y}^{\top}\mathbf{H}^{-1}\mathbf{H}^{-1}\mathbf{y}}{tr(\mathbf{H}^{-1})}\right) \qquad (6)
\end{aligned}$$

Now consider the score $U_{\sigma_e^2} = \mathbf{0}$. Using the same quantity $\mathbf{V}^{-1} = \frac{\mathbf{H}^{-1}}{\sigma_e^2}$, we have:

$$
\begin{aligned}
tr(\mathbf{V}^{-1}) &= \mathbf{y}^\top \mathbf{V}^{-\mathbf{1}} \mathbf{V}^{-\mathbf{1}} \mathbf{y} \\
tr(\mathbf{H}^{-1}) &= \frac{\mathbf{y}^\top \mathbf{H}^{-\mathbf{1}} \mathbf{H}^{-\mathbf{1}} \mathbf{y}}{\sigma_e^2}
\end{aligned} \tag{7}
$$

Plugging Supplementary Equation (7) into Supplementary Equation (6) and substituting H back with V, we have:

$$
\begin{aligned}
n\sigma_e^2 &= \mathbf{y}^\top \mathbf{H}^{-\mathbf{1}} \mathbf{y} \\
\Rightarrow \sigma_e^2 &= \frac{\mathbf{y}^\top \mathbf{H}^{-\mathbf{1}} \mathbf{y}}{n} = \frac{\mathbf{y}^\top \mathbf{V}^{-\mathbf{1}} \mathbf{y} \sigma_e^2}{n}.
\end{aligned} \tag{8}
$$

Finally, to express σ_e^2 as a function of BLUP, we use the the Woodbury formula to get:

$$
\begin{aligned}
\mathbf{V}^{-\mathbf{1}}\mathbf{y} &= \left(\frac{1}{\sigma_e^2}\mathbf{I} - \frac{1}{\sigma_e^4}\mathbf{X}\left(\frac{1}{\sigma_g^2}\mathbf{I} + \mathbf{X}^\top\mathbf{X}\frac{1}{\sigma_e^2} \right)^{-1} \mathbf{X}^\top \right) \mathbf{y} \\
&= \frac{1}{\sigma_e^2}\mathbf{y} - \frac{1}{\sigma_e^2}\mathbf{X}\left(\frac{1}{\sigma_g^2}\mathbf{I} + \mathbf{X}^\top\mathbf{X}\frac{1}{\sigma_e^2} \right)^{-1} \mathbf{X}^\top\mathbf{y}\frac{1}{\sigma_e^2} \\
&= \frac{1}{\sigma_e^2}\mathbf{y} - \frac{1}{\sigma_e^2}\mathbf{X}\left(\frac{\sigma_e^2}{\sigma_g^2}\mathbf{I} + \mathbf{X}^\top\mathbf{X} \right)^{-1} \mathbf{X}^\top\mathbf{y} \\
&= \frac{1}{\sigma_e^2}(\mathbf{y} - \mathbf{X})
\end{aligned} \tag{9}
$$

where $\tilde{\beta}$ is the BLUP estimate: $\left(\frac{\sigma_e^2}{\sigma_g^2}\mathbf{I} + \mathbf{R}\right)^{-1}\mathbf{S}$. Plugging Supplementary Equation (9) into Supplementary Equation (8) gives us the expression for updating σ_e^2 using the BLUP:

$$
\sigma_e^2 = \frac{\mathbf{y}^\top(\mathbf{y} - \mathbf{X})}{n}.
$$

Updating equation for σ_g^2

Define $\mathbf{W} = \left(\mathbf{I} + \frac{\sigma_g^2}{\sigma_e^2}\mathbf{R}\right)$. Then we have $\mathbf{W} - \mathbf{I} = \frac{\mathbf{R}\sigma_g^2}{\sigma_e^2}$. The LHS of the score equation can be rewritten, using the Woodbury identity matrix, we have:

$$
\begin{aligned}
tr(\mathbf{X}^\top \mathbf{V}^{-1}\mathbf{X}) &= tr\left(\mathbf{X}^\top \left(\frac{1}{\sigma_e^2}\left(\mathbf{I} - \frac{\sigma_g^2}{\sigma_e^2}\mathbf{X}\mathbf{W}^{-1}\mathbf{X}^\top\right)\right)\mathbf{X}\right) \\
&= tr\left(\frac{\mathbf{R}}{\sigma_e^2} - \frac{\sigma_g^2}{\sigma_e^2}\mathbf{R}\mathbf{W}^{-1}\frac{\mathbf{R}}{\sigma_e^2}\right) \\
&= tr\left(\left(\frac{\mathbf{R}\sigma_g^2}{\sigma_e^2} - \frac{\mathbf{R}\sigma_g^2}{\sigma_e^2}\mathbf{W}^{-1}\frac{\mathbf{R}\sigma_g^2}{\sigma_e^2}\right)\frac{1}{\sigma_g^2}\right) \\
&= tr\left(\frac{1}{\sigma_g^2}(\mathbf{W} - \mathbf{I} - (\mathbf{W} - \mathbf{I})\mathbf{W}^{-1}(\mathbf{W} - \mathbf{I}))\right) \\
&= tr\left(\frac{1}{\sigma_g^2}(\mathbf{I} - \mathbf{W}^{-1})\right) \\
&= \frac{p - tr(\mathbf{W}^{-1})}{\sigma_g^2}.
\end{aligned} \tag{10}
$$

The RHS of the score equation can be rewritten as:

$$
\mathbf{y}^\top \mathbf{V}^{-1}\mathbf{X}\mathbf{X}^\top \mathbf{V}^{-1}\mathbf{y} = \frac{\mathbf{y}^\top \mathbf{V}^{-1}\mathbf{X}\sigma_g^2\sigma_g^2\mathbf{X}^\top \mathbf{V}^{-1}\mathbf{y}}{\sigma_g^4} = \frac{\tilde{}^\top\tilde{}}{\sigma_g^4}. \tag{11}
$$

Equating Supplementary Equation (10) and Supplementary Equation (11) leads us to have two equivalent updating equations for σ_g^2:

$$
\sigma_g^2 = \frac{\tilde{}^\top\tilde{} + \sigma_e^2 tr(\mathbf{W}^{-1})}{p} \quad \text{or} \quad \sigma_g^2 = \frac{\tilde{}^\top\tilde{}}{p - tr(\mathbf{W}^{-1})}.
$$

In summary, we have shown that the score equations of (σ_g^2, σ_e^2) derived from the likelihood in Supplementary Equation (1) are identical to the score equations based on Supplementary Equation (5), giving rise to our updating equations in the HEELS estimation procedure.

Supplementary Note 4

When sample variance is not known, we can approximate $\mathbf{y}^\top\mathbf{y}/n$ in two ways. Suppose the effect sizes and standard errors are both provided in the summary statistics. For a given SNP j, consider the marginal model used in the association studies, $\mathbf{y} = \mathbf{X}_{\cdot j}\alpha + \gamma$, where, without loss of generality, we denote by α and γ the marginal association effect size and the error term in the marginal model, respectively, which

are to be distinguished from and ε defined in the joint model. $\mathbf{X}_{\cdot j}$ is the j-th column of $\mathbf{X}$ and has been standardized to have variance 1.

$$
\begin{aligned}
\frac{\mathbf{y}^\top \mathbf{y}}{n} &= \frac{1}{n}(\mathbf{X}_{\cdot j}\alpha + \gamma)^\top (\mathbf{X}_{\cdot j}\alpha + \gamma) \\
&= \frac{1}{n}(\alpha^2 \mathbf{X}_{\cdot j}^\top \mathbf{X}_{\cdot j} + 2\alpha \mathbf{X}_{\cdot j}^\top \gamma + \gamma^\top \gamma) \\
&= \alpha^2 + \frac{\gamma^\top \gamma}{n} \qquad \text{(independence between genotype and the error term)} \\
&= \alpha^2 + \sigma_\gamma^2 \approx \frac{1}{p}\sum_{j=1}^{p}(\widehat{\alpha}_j^2 + n\widehat{SE}_j^2),
\end{aligned}
$$

where we use the mean estimator in the last step. σ_γ^2 denotes the residual variance in the marginal model. Alternatively, we can assume a unit-variance phenotype and use the standardized effect sizes to estimate $\hat{h}^2_{HEELS}$. We use this second approximation in practice, as it is more generally applicable, especially when only the Z-scores or p-values are provided in the summary statistics. Accordingly, we normalize the variance component estimates at each iteration such that $\sigma_g^2 + \sigma_e^2 = 1$.

Supplementary Note 5

In this section, we demonstrate that the HEELS procedure can be viewed as an EM algorithm. We proceed by first formulating the EM algorithm using the same model as the one we used for deriving HEELS. Then we connect it to the HEELS estimation procedure by showing its equivalence to the REML estimating equations. In essence, EM maximizes the likelihood of the complete or augmented data, which in our case is the phenotype vector and the unobserved random effects, $(\mathbf{y}, \beta)$. The incomplete data, on the other hand, is the observed phenotype vector, $\mathbf{y}$.

EM iterates between updating the random effects using its expected conditional mean, and updating the variance components using the maximizers of the log-likelihood based on the joint density. Given the joint distribution of the complete data specified in Supplementary Equation (4), the conditional distribution of the random effects is:

$$
\beta|\mathbf{y} \sim MVN(\sigma_g^2 \mathbf{X}^\top \mathbf{V}^{-\mathbf{1}}\mathbf{y}, \sigma_g^2 \mathbf{I}_p - \sigma_g^4 \mathbf{X}^\top \mathbf{V}^{-\mathbf{1}}\mathbf{X}).
$$

Hence, the conditional expected value of the random effects is $\mathbb{E}(\beta|\mathbf{y}) = \sigma_g^2 \mathbf{X}^\top \mathbf{V}^{-\mathbf{1}}\mathbf{y}$. The log-likelihood based on the complete data is,

$$
l(\sigma_g^2, \sigma_e^2; \mathbf{y},) \propto -\frac{1}{2}\left\{ p \cdot log(\sigma_g^2) + n \cdot log(\sigma_e^2) + \frac{\top}{\sigma_g^2} + \frac{(\mathbf{y} - \mathbf{X})^\top (\mathbf{y} - \mathbf{X})}{\sigma_e^2} \right\}, \tag{12}
$$

and the maximizers of which can be easily derived as,

$$\sigma_g^2 = \frac{^\top}{p} \quad \text{and} \quad \sigma_e^2 = \frac{(\mathbf{y}-\mathbf{X}\beta)^\top(\mathbf{y}-\mathbf{X}\beta)}{n}. \tag{13}$$

The maximization step uses the conditional expectation $\mathbb{E}(\beta|\mathbf{y})$ in place of the true unknown random effects β in (13). To summarize, the EM procedure for estimating the variance components alternates between the following two steps,

- At the E-step, we update the random effects using the incomplete data and the current value of the variance components from the M-step,

$$^{(t)} = \sigma_g^{2(t)}\mathbf{X}^\top\mathbf{V}^{-\mathbf{1}}\mathbf{y}. \tag{14}$$

- At the M-step, we update the variance components using the complete data and the conditional expectation of the random effects from the E-step,

$$\sigma_g^{2(t)} = \frac{\mathbb{E}(^\top|\mathbf{y})}{p}\Bigg|_{\beta^{(t)}} = \frac{1}{p}\left(\sigma_g^{4(t-1)}\mathbf{y}^\top\mathbf{V}^{-\mathbf{1}}\mathbf{X}\mathbf{X}^\top\mathbf{V}^{-\mathbf{1}}\mathbf{y} + tr(\sigma_g^{2(t-1)}\mathbf{I}_p - \sigma_g^{4(t-1)}\mathbf{X}^\top\mathbf{V}^{-\mathbf{1}}\mathbf{X})\right) \tag{15}$$

$$\sigma_e^{2(t)} = \frac{\mathbb{E}((\mathbf{y}-\mathbf{X})^\top(\mathbf{y}-\mathbf{X})|\mathbf{y})}{n}\Bigg|_{\beta^{(t)}} = \frac{1}{n}\left(\sigma_e^{4(t-1)}\mathbf{y}^\top\mathbf{V}^{-\mathbf{2}}\mathbf{y} + tr(\sigma_e^{2(t-1)}\mathbf{I}_n - \sigma_e^{4(t-1)}\mathbf{V}^{-1})\right) \tag{16}$$

  where the second equality in both equation (15) and (16) follows from the conditional distributional form of $\beta|\mathbf{y}$ and the quadratic form theory.

To see the connections between the EM algorithm and our estimation procedure, note that the updating equations (15) and (16) are fixed-point iterations which lead to the same solutions as the estimating equations solved by HEELS.

$$\begin{aligned}
p\sigma_g^2 &= \sigma_g^4\mathbf{y}^\top\mathbf{V}^{-\mathbf{1}}\mathbf{X}\mathbf{X}^\top\mathbf{V}^{-\mathbf{1}}\mathbf{y} + tr(\sigma_g^2\mathbf{I}_p - \sigma_g^4\mathbf{X}^\top\mathbf{V}^{-\mathbf{1}}\mathbf{X}) \\
0 &= \mathbf{y}^\top\mathbf{V}^{-\mathbf{1}}\mathbf{X}\mathbf{X}^\top\mathbf{V}^{-\mathbf{1}}\mathbf{y} - tr(\mathbf{X}^\top\mathbf{V}^{-\mathbf{1}}\mathbf{X}) && \text{(same as equation (6))} \\
n\sigma_e^2 &= \sigma_e^4\mathbf{y}^\top\mathbf{V}^{-\mathbf{2}}\mathbf{y} + tr(\mathbf{I}_n - \sigma_e^2\mathbf{V}^{-1}) \\
0 &= \mathbf{y}^\top\mathbf{V}^{-\mathbf{2}}\mathbf{y} - tr(\mathbf{V}^{-1}). && \text{(same as equation (7))}
\end{aligned}$$

Lastly, the updating equation in (14) coincide with the updating equation for β in HEELS. Therefore, the HEELS estimation procedure can be viewed as an EM algorithm under the same model. Notably, HEELS has several nice properties which are also features of the EM algorithm, such as guarantee of convergence under fairly unrestricted conditions[34,35]. Viewing the HEELS algorithm as an EM is a helpful step for extending our method to incorporate sparse or heterogeneous effect size variances. For example, we can modify the complete likelihood function to incorporate flexible weighting of SNPs; moreover, we

can introduce latent variables to indicate the markers' null vs non-null effects and expand the E-step to update this latent variable together with .

Supplementary Note 6

Here we explain the similarities and differences between the GRE estimator and the HESS estimator of SNP-heritability. First of all, both the GRE estimator and the HESS estimator have a closed-form expression and are functions of the marginal association statistics and in-sample LD. While GRE is a genome-wide h^2_{SNP} estimator, it assumes independent and additive chromosome-wide heritabilities. Hence, the form of the *regional* GRE estimator in fact coincides with that of the HESS estimator when LD is estimated in-sample,

$$\hat{h}^2_{GRE} = \sum_{c=1}^{22} \frac{n\hat{\beta}_c^\top \Sigma_c^\dagger \hat{\beta}_c - p_c}{n - p_c} \quad \text{, where } c \text{ signifies chromosome;}$$

$$\hat{h}^2_{HESS_k} = \frac{n\hat{\beta}_k^\top \Sigma_k^\dagger \hat{\beta}_k - p_k}{n - p_k} \quad \text{, where } k \text{ signifies region.}$$

Note that throughout this section, we adopt the notations used in the GRE and HESS papers: $\Sigma_c^\dagger$ and $\Sigma_k^\dagger$ are the pseudo-inverse of the empirical chromosome-wide or regional LD matrices. The definitions of Σ involves the scaling by $1/n$, which are to be distinguished from the definition of $\mathbf{R}$ in our work. $\hat{\beta}_c$ and $\hat{\beta}_k$ are *unstandardized* marginal association statistics from OLS, *i.e.*, $\hat{\beta}_c = \mathbf{X}^\top \mathbf{y}/n$, which are different from the *standardized* marginal association statistics $\mathbf{S}$ in HEELS.

GRE and HESS can both be viewed as principal-component regression (PCR) based estimators. In other words, both of these two estimators are weighted summations of the squared projections of GWAS effect sizes onto the eigenvectors of the LD matrix. Denote the singular value decomposition of $\mathbf{X}$ by $\mathbf{X} = \mathbf{U}\Lambda\mathbf{V}^\top$. Then the covariance matrix can be written as $\mathbf{X}^\top\mathbf{X} = \mathbf{V}\Lambda^2\mathbf{V}^\top$. We can project $\mathbf{X}$ onto a lower-dimensional space using the eigenvectors of its covariance matrix:

$$\mathbf{y} = \mathbf{X}\beta_J + \varepsilon = \underbrace{\mathbf{XV}}_{\mathbf{Z}}\underbrace{\mathbf{V}^\top\beta_J}_{\gamma} + \varepsilon = \mathbf{Z}\gamma + \varepsilon,$$

where we use β_J to denote the true joint effect size, to be distinguished from the true marginal effect size $\beta_M = \Sigma\beta_J$. In this analysis, we consider the marginal association statistics generally, so β_M can refer to either $\hat{\beta}_c$ in GRE or $\hat{\beta}_k$ in HESS. Assuming unit-variance phenotypes or standardized effect sizes, the PCR-estimator for heritability when the top r principal components are used is,

$$\hat{h}^2_{PCR_r} = 1 - \hat{\sigma}_e^2 = 1 - \frac{(\mathbf{y} - \mathbf{Z_r}\gamma_\mathbf{r})^\top(\mathbf{y} - \mathbf{Z_r}\gamma_\mathbf{r})}{n - r},$$

where the projected genotype matrix and effect size are $\mathbf{Z}_r = \mathbf{X}\mathbf{V_r}$ and $\gamma_r = \mathbf{V}_r^\top\beta_J$. Notably,

$$
\begin{aligned}
\hat{\sigma}_e{}^2 &= \frac{(\mathbf{y}-\mathbf{Z_r}\gamma_\mathbf{r})^\top(\mathbf{y}-\mathbf{Z_r}\gamma_\mathbf{r})}{n-r} \\
&= \frac{\mathbf{y}^\top\mathbf{y}-2\mathbf{y}^\top\mathbf{Z_r}\gamma_r+\gamma_r^\top\mathbf{Z_r}^\top\mathbf{Z_r}\gamma_r}{n-r} \\
&= \frac{\mathbf{y}^\top\mathbf{y}-2\mathbf{y}^\top\mathbf{X}\mathbf{V_r}\mathbf{V_r}^\top\beta_J+\beta_J^\top\mathbf{V_r}\mathbf{V_r}^\top\mathbf{X}^\top\mathbf{X}\mathbf{V_r}\mathbf{V_r}^\top\beta_J}{n-r} \\
&= \frac{n-2n\beta_M^\top\mathbf{V_r}\mathbf{V_r}^\top\beta_J+\beta_J^\top n\Sigma\beta_J}{n-r} && \text{(orthogonality of } \mathbf{V}_r\text{)} \\
&= \frac{n-n\beta_J^\top\Sigma\beta_J}{n-r} && \text{(use } \beta_M=\Sigma\beta_J\text{)} \\
&= \frac{n-n\beta_M^\top\Sigma^{-1}\beta_M}{n-r} && \text{(use } \beta_J=\Sigma^{-1}\beta_M\text{)} \\
1-\hat{\sigma}_e^2 &= \frac{n\beta_M^\top\Sigma^{-1}\beta_M-r}{n-r} && (17)
\end{aligned}
$$

Therefore, $\hat{h}^2_{PCR_r}$ is precisely the GRE estimator when $r=\min(n,p)=rank(\mathbf{X})$ and is the HESS estimator with truncated SVD using the top r eigenvectors, when Σ^{-1} is approximated by $\mathbf{V}_r\Lambda_r^{-2}\mathbf{V_r}^\top$.

An important similarity between the GRE estimator and the HESS estimator is that they are both robust to different underlying genetic architectures. HESS is unbiased as it models the causal effects as fixed; GRE is consistent as it allows arbitrary and SNP-specific variance of causal effects. Moreover, both methods require $n >> p$. The HESS estimator is applied at the local level to independent loci, so it does not require ultra-large sample size. The GRE estimator requires biobank-scale data as it is applied to the whole genome. For both estimators, the requirement for large sample size arises from the asymptotics used in deriving the unbiased estimator. The variance of both estimators are similarly developed based on quadratic form theory.

The two estimators differ in several aspects. First, GRE is derived under the framework of random effects whereas HESS assumes the genetic effects are fixed. In other words, the estimands or the target quantity h^2_{SNP} defined by these two methods are slightly different:

$$
\text{Random effects model (adopted by GRE): } h^2_{SNP}=\mathbb{E}(\beta^\top\Sigma\beta)=\sum_{j=1}^{p}\sigma_j^2;
$$
$$
\text{Fixed effects model (adopted by HESS): } h^2_{SNP}=\beta^\top\Sigma\beta.
$$

where Σ is the population LD and σ_j^2 is the marker-specific effect size variance for SNP j, assumed to be any arbitrary positive value. GRE can be viewed as a generalized method-of-moment estimator, as it was derived by equating the population and the sample moment $\beta_M^\top\Sigma^{-1}\beta_M$ (see equation (4) in the Supplementary notes of[20] and the expression right above it). Note that the moment equating step assumes

conditioning both of the effect size and genotype, the estimator may be considered as a fixed-effect heritability estimator.

Second, GRE uses in-sample LD information and thus does not require regularization of the LD matrix, whereas HESS is designed to account for the LD structure using a reference panel and thus regularizes out-of-sample LD via truncated SVD. When LD is estimated in-sample and no LD regularization is applied, *i.e.* $r = rank(\mathbf{X})$, the two estimators coincide completely. Indeed, we observed in simulations that the estimates from HESS approach the estimates from GRE as LD regularization lessens (Supplementary Figure 2).

Supplementary Note 7

To derive the asymptotic variance of the HEELS estimator, we start by first deriving the information matrix for $\ell(\mathbf{y};\sigma_g^2,\sigma_e^2)$. Given that $U_{\sigma_g^2}(\mathbf{y}) = -\frac{1}{2}tr(\mathbf{X}^\top\mathbf{V}^{-1}\mathbf{X}) + \frac{1}{2}(\mathbf{X}^\top\mathbf{V}^{-1}\mathbf{y})^\top(\mathbf{X}^\top\mathbf{V}^{-1}\mathbf{y})$, we have,

$$\begin{aligned}
-\mathbb{E}\left(\frac{\partial U_{\sigma_g^2}(\mathbf{y})}{\partial \sigma_g^2}\right) &= -\frac{1}{2}tr(\mathbf{V}^{-1}\mathbf{X}\mathbf{X}^\top\mathbf{V}^{-1}\mathbf{X}\mathbf{X}^\top) + \mathbb{E}(\mathbf{y}^\top\mathbf{V}^{-1}\mathbf{X}\mathbf{X}^\top\mathbf{V}^{-1}\mathbf{X}\mathbf{X}^\top\mathbf{V}^{-1}\mathbf{y})\\
&= -\frac{1}{2}tr(\mathbf{V}^{-1}\mathbf{X}\mathbf{X}^\top\mathbf{V}^{-1}\mathbf{X}\mathbf{X}^\top) + tr(\mathbf{V}^{-1}\mathbf{X}\mathbf{X}^\top\mathbf{V}^{-1}\mathbf{X}\mathbf{X}^\top\mathbf{V}^{-1}\mathbf{V})\\
&= \frac{1}{2}tr(\mathbf{V}^{-1}\mathbf{X}\mathbf{X}^\top\mathbf{V}^{-1}\mathbf{X}\mathbf{X}^\top).
\end{aligned}$$

Given that $U_{\sigma_e^2}(\mathbf{y}) = -\frac{1}{2}tr(\mathbf{V}^{-1}) + \frac{1}{2}\mathbf{y}^\top\mathbf{V}^{-1}\mathbf{V}^{-1}\mathbf{y}$, we have,

$$\begin{aligned}
-\mathbb{E}\left(\frac{\partial U_{\sigma_e^2}(\mathbf{y})}{\sigma_e^2}\right) &= -\frac{1}{2}tr(\mathbf{V}^{-2}) + \mathbb{E}(\mathbf{y}^\top\mathbf{V}^{-2}\mathbf{V}^{-1}\mathbf{y})\\
&= -\frac{1}{2}tr(\mathbf{V}^{-2}) + tr(\mathbf{V}^{-2}\mathbf{V}^{-1}\mathbf{V})\\
&= \frac{1}{2}tr(\mathbf{V}^{-2}).
\end{aligned}$$

The cross-term of the information matrix can be derived as:

$$\begin{aligned}
-\mathbb{E}\left(\frac{\partial U_{\sigma_g^2}(\mathbf{y})}{\partial \sigma_e^2}\right) &= -\frac{1}{2}tr(\mathbf{V}^{-1}\mathbf{X}\mathbf{X}^\top\mathbf{V}^{-1}) + \mathbb{E}(\mathbf{y}^\top\mathbf{V}^{-1}\mathbf{X}\mathbf{X}^\top\mathbf{V}^{-1}\mathbf{V}^{-1}\mathbf{y})\\
&= -\frac{1}{2}tr(\mathbf{V}^{-1}\mathbf{X}\mathbf{X}^\top\mathbf{V}^{-1}) + tr(\mathbf{V}^{-1}\mathbf{X}\mathbf{X}^\top\mathbf{V}^{-1}\mathbf{V}^{-1}\mathbf{V})\\
&= \frac{1}{2}tr(\mathbf{V}^{-1}\mathbf{X}\mathbf{X}^\top\mathbf{V}^{-1}).
\end{aligned}$$

Therefore, for true values of the variance components σ_e^2, σ_g^2 and positive definite $\mathbf{V}$, the information matrix is:

$$I(\sigma_e^2, \sigma_g^2; \mathbf{X}, \mathbf{y}) = \frac{1}{2} \begin{bmatrix} tr(\mathbf{V^{-2}}) & tr(\mathbf{V^{-1}XX^{\top}V^{-1}}) \\ tr(\mathbf{V^{-1}XX^{\top}V^{-1}}) & tr(\mathbf{V^{-1}XX^{\top}V^{-1}XX^{\top}}) \end{bmatrix}.$$

Using properties of trace and the identity of $\mathbf{W} := \frac{\sigma_e^2}{\sigma_g^2}\mathbf{I} + \mathbf{R}$[33], we can rewrite the information matrix using only the summary statistics and in-sample LD as the following:

$$I(\sigma_e^2, \sigma_g^2; \mathbf{S}, \mathbf{R}) = \frac{1}{2} \begin{bmatrix} \frac{n-p}{\sigma_\mathbf{e}^4} + \frac{\mathbf{1}}{\sigma_\mathbf{g}^4} tr(\mathbf{W^{-2}}) & \frac{\mathbf{1}}{\sigma_\mathbf{g}^4} tr(\mathbf{W^{-1}}) - \frac{\sigma_\mathbf{e}^2}{\sigma_\mathbf{g}^6} tr(\mathbf{W^{-2}}) \\ \frac{\mathbf{1}}{\sigma_\mathbf{g}^4} tr(\mathbf{W^{-1}}) - \frac{\sigma_\mathbf{e}^2}{\sigma_\mathbf{g}^6} tr(\mathbf{W^{-2}}) & \frac{p}{\sigma_g^4} - \frac{\mathbf{2}\sigma_\mathbf{e}^2}{\sigma_\mathbf{g}^6} tr(\mathbf{W^{-1}}) + \frac{\sigma_\mathbf{e}^4}{\sigma_\mathbf{g}^8} tr(\mathbf{W^{-2}}) \end{bmatrix} \tag{18}$$

Supplementary Note 8

In this section, we provide details on the asymptotic variance of h^2_{HEELS} when the LD matrix has a low dimensional representation. We only describe the scenario where the Banded + LR structure is employed, as the other strategies are special cases of this general setting. Let $\mathbf{W} := \frac{\sigma_e^2}{\sigma_g^2}\mathbf{I} + \mathbf{R}$ be the working matrix, as defined before. Suppose we have obtained the approximation form of the LD matrix, denoted by $\mathbf{R} \approx \mathbf{R}_b + \mathbf{U}_r \Lambda_r \mathbf{U}^{\top}$, we then have an approximation form of the working matrix $\mathbf{W} \approx \mathbf{W}_b + \mathbf{U}_r \Lambda_r \mathbf{U}^{\top}$ with $\mathbf{W}_b = \mathbf{R}_b + \frac{\sigma_e^2}{\sigma_g^2} I$. For any given matrices $\mathbf{Y} \in \mathbb{R}^{p \times p}, \mathbf{Z} \in \mathbb{R}^{p \times r}, \Lambda \in \mathbb{R}^{r \times r}$, we define a mapping,

$$f : \mathbb{R}^{p \times p} \times \mathbb{R}^{p \times r} \times \mathbb{R}^{r \times r} \longrightarrow \mathbb{R}^{p \times p}$$
$$f(\mathbf{Y}, \mathbf{Z}, \Lambda) = \mathbf{Y} - \mathbf{YZ}(\Lambda^{-1} + \mathbf{Z}^{\top}\mathbf{YZ})^{-1}\mathbf{Z}^{\top}\mathbf{Y}^{\top}.$$

This mapping corresponds to the Woodbury matrix inverse formula, *i.e.* $\mathbf{W}^{-1} = f(\mathbf{W}_b, \mathbf{U}_r, \Lambda_r)$ if $\mathbf{W} = \mathbf{W}_b + \mathbf{U}_r \Lambda_r \mathbf{U}^{\top}$. We can apply this mapping again to express the squared term, $\mathbf{W}^{-2}$,

$$\begin{aligned} \mathbf{W}^{-2} &\approx (\mathbf{W}_b^{\top}\mathbf{W}_b + 2\mathbf{W}_b^{\top}\mathbf{U}_r\Lambda_r\mathbf{U}_r^{\top} + \mathbf{U}_r\Lambda_r^2\mathbf{U}_r^{\top})^{-1} \\ &= (\mathbf{V} + \mathbf{U}_r\Lambda_r^2\mathbf{U}_r^{\top})^{-1} \qquad \text{(Let } \mathbf{V} = \mathbf{W}_b^{\top}\mathbf{W}_b + 2\mathbf{W}_b^{\top}\mathbf{U}_r\Lambda_r\mathbf{U}_r^{\top}) \\ &\approx \mathbf{V}^{-1} - \mathbf{V}^{-1}\mathbf{U}_r(\Lambda_r^{-2} + \mathbf{U}_r^{\top}\mathbf{V}^{-1}\mathbf{U}_r)^{-1}\mathbf{U}_r^{\top}\mathbf{V}^{-1} \\ &= f(\mathbf{V}^{-1}, \mathbf{U}_r, \Lambda_r^2). \end{aligned}$$

Furthermore, we note that $\mathbf{V}^{-1} = (\mathbf{W}_b + 2\mathbf{U}_r\Lambda_r\mathbf{U}_r^{\top})^{-1}\mathbf{W_b}^{-1} = f(\mathbf{W}_b, \mathbf{U}_r, 2\Lambda_r)\mathbf{W_b}^{-1}$. Hence, both $\mathbf{W}^{-1}$ and $\mathbf{W}^{-2}$ can be expressed as functions of the approximating elements of the LD matrix:

$$\mathbf{W}^{-1} = f(\mathbf{W}_b, \mathbf{U}_r, \Lambda_r) \tag{19}$$
$$\mathbf{W}^{-2} = f(f(\mathbf{W}_b, \mathbf{U}_r, 2\Lambda_r)\mathbf{W}_b^{-1}, \mathbf{U}_r, \Lambda_r^2). \tag{20}$$

Therefore, in cases where the LD matrix has been approximated by the sum of a banded matrix and a

low-rank matrix, we can still estimate the variance of the HEELS estimator without incurring additional computational costs.

Supplementary Note 9

In this section, we demonstrate how our estimator is equivalent to the most efficient estimator under the Generalized Method of Moments (GMM) framework. Given the same modeling of the phenotypes as is defined in the main text, but without the distributional assumption, the estimating equations for the method of moments estimators take the form of,

$$\mathbf{y}^\top \mathbf{A}_1 \mathbf{y} = tr(\mathbf{A}_1 \mathbf{X}\mathbf{X}^\top)\sigma_g^2 + tr(\mathbf{A}_1)\sigma_e^2 \tag{21}$$

$$\mathbf{y}^\top \mathbf{A}_2 \mathbf{y} = tr(\mathbf{A}_2 \mathbf{X}\mathbf{X}^\top)\sigma_g^2 + tr(\mathbf{A}_2)\sigma_e^2, \tag{22}$$

where $\mathbf{A_1}, \mathbf{A_2}$ are two symmetric non-negative definite matrices of dimension $n \times n$, used as weights. From the theory of method of moments, the choice of $\mathbf{A}$ will not affect the unbiasedness of σ_g^2, σ_e^2, but can affect the statistical efficiency of the estimators. A common criterion for selecting the optimal weight matrices is to minimize the expected squared error (*i.e.* the difference between population and sample moments), which is called the "Minimal Norm Quadratic Unbiased Estimation (MINQUE)" criterion[19]. For $l = 1, 2$, we solve the following objective function,

$$\min E\left[\left(\mathbf{y}^\top \mathbf{A_l} \mathbf{y} - tr(\mathbf{A}_l \mathbf{X}\mathbf{X}^\top)\sigma_g^2 - tr(\mathbf{A}_l)\sigma_e^2\right)^2\right], \tag{23}$$

which leads to the Best Quadratic Unbiased Estimator (BQUE). The optimal weights that minimize the objective in Supplementary Equation (23) are:

$$\mathbf{A}_1^* = \mathbf{V}^{-1}\mathbf{X}\mathbf{X}^\top\mathbf{V}^{-\mathbf{1}}; \quad \mathbf{A}_2^* = \mathbf{V}^{-\mathbf{1}}\mathbf{V}^{-\mathbf{1}}.$$

Since both $\mathbf{A}_1^*$ and $\mathbf{A}_2^*$ involve unknown population parameters through $\mathbf{V}$, the estimators of σ_g^2, σ_e^2 can only be obtained via an iterative procedure[19]. We next demonstrate how our HEELS estimator minimizes the expected squared error and thus meets the MINQUE criterion. It suffices to show that Supplementary Equations (2) and (3) coincide with the moment matching equations in Supplementary Equations (21) and (22). With the optimal weights, $\mathbf{A}_1^*, \mathbf{A}_2^*$, Supplementary Equation (21) can be equated with Supplementary

Equation (2) as:

$$\begin{aligned}
\mathbf{y}^\top(\mathbf{V}^{-1}\mathbf{X}\mathbf{X}^\top\mathbf{V}^{-1})\mathbf{y} &= tr(\mathbf{V}^{-1}\mathbf{X}\mathbf{X}^\top\mathbf{V}^{-1}\mathbf{X}\mathbf{X}^\top)\sigma_g^2 + tr(\mathbf{V}^{-1}\mathbf{X}\mathbf{X}^\top\mathbf{V}^{-1})\sigma_e^2\\
(\mathbf{X}^\top\mathbf{V}^{-1}\mathbf{y})^\top(\mathbf{X}^\top\mathbf{V}^{-1}\mathbf{y}) &= tr(\mathbf{V}^{-1}\mathbf{X}\mathbf{X}^\top\mathbf{V}^{-1}(\mathbf{X}\mathbf{X}^\top\sigma_g^2\mathbf{I}_n + \sigma_e^2\mathbf{I}_n))\\
&= tr(\mathbf{V}^{-1}\mathbf{X}\mathbf{X}^\top\mathbf{V}^{-1}\mathbf{V})\\
&= tr(\mathbf{X}^\top\mathbf{V}^{-1}\mathbf{X}).
\end{aligned}$$

Analogously, Supplementary Equation (22) can be equated with Supplementary Equation (3) as:

$$\begin{aligned}
\mathbf{y}^\top(\mathbf{V}^{-1}\mathbf{V}^{-1})\mathbf{y} &= tr(\mathbf{V}^{-1}\mathbf{V}^{-1}\mathbf{X}\mathbf{X}^\top)\sigma_g^2 + tr(\mathbf{V}^{-1}\mathbf{V}^{-1})\sigma_e^2\\
(\mathbf{V}^{-1}\mathbf{y})^\top(\mathbf{V}^{-1}\mathbf{y}) &= tr(\mathbf{V}^{-1}\mathbf{V}^{-1}(\mathbf{K}\boldsymbol{\sigma}_{\mathbf{g}}^{\mathbf{2}}\mathbf{I}_n + \sigma_e^2\mathbf{I}_n))\\
&= tr(\mathbf{V}^{-1}).
\end{aligned}$$

Zhou (2017) showed that the heritability estimator of LDSC (without or with known population structure) can be viewed as a generalized method of moments estimator under the GMM framework, but with sub-optimal weights[19]. In other word, the statistical efficiency of LDSC is lower than that of BQUE. As we have established the equivalence of efficiency between our HEELS estimator and the BQUE above, we conclude that HEELS is statistically more efficient than LDSC, as is confirmed by the simulation results.

Supplementary Note 10

In two of our proposed "Banded + LR" LD approximation strategies, "Seq_Band_LR" and "PSD_Band_LR", we impose the PSD assumption, which helps to both 1) reduce the computational burden of solving the LD decomposition, and 2) improve the efficiency of our heritability estimation algorithm.

First, it can reduce the computational burden of searching for the low-dimensional representation. Consider the following two minimization problems,

$$\tilde{\mathbf{R}}^b, \tilde{\mathbf{R}}^r = \arg\min_{\mathbf{R}^b, \mathbf{R}^r \in \mathbb{R}^{p\times p}} ||\mathbf{R} - \mathbf{R}^b - \mathbf{R}^r||_F^2 \tag{24}$$

$$\tilde{\mathbf{L}}^b, \tilde{\mathbf{U}}^r = \arg\min_{\mathbf{L}^b, \mathbf{U}^r \in L_p(\mathbb{R})} ||\mathbf{R} - \mathbf{L}^{b\top}\mathbf{L}^b - \mathbf{U}^{r\top}\mathbf{U}^r||_F^2 \tag{25}$$

where $L_p(\mathbb{R})$ denotes the set of $p \times p$ lower triangular matrices with real entries. The objective of both of these optimization procedures is to minimize the squared Frobenius norm of the error matrix. However, optimizing over the Cholesky factors of the banded and the low-rank components of the representation in Supplementary Equation (25) leads to fewer parameters than optimizing all elements of the banded and low-rank matrices in Supplementary Equation (24). The reduction of computational burden is substantial when p is large.

Another benefit of using the PSD assumption is that the Cholesky factors, $\tilde{\mathbf{L}}^b, \tilde{\mathbf{U}}^r$, can be directly used to compute the inverse of the LD matrix in the HEELS estimation procedure, without incurring additional cost. To be specific, the major computational burden of the HEELS estimating procedure lies in the evaluation of $\mathbf{W^{(t)-1}}$ and $tr(\mathbf{W^{(t)-1}})$. In the absence of LD approximation, we compute the Cholesky factors of the working matrix $\mathbf{W}^{(t)}$ once per iteration, and these factors are used to calculate $\mathbf{W^{(t)-1}S}$ and $tr(\mathbf{W^{(t)-1}})$ respectively. The PSD guarantee of the solutions lessens the computational cost of these two steps significantly by circumventing the need to compute the Cholesky factors of the working matrix $\mathbf{W}^{(t)}$ at every iteration.

Suppose the LD matrix has been approximated as $\mathbf{R} \approx \mathbf{R}_b + \mathbf{U}_r \Lambda_r \mathbf{U}_r^\top$. We apply the Sherman-Morrison-Woodbury formula to circumvent the inversion of $\mathbf{W}^{(t)}$ as the following,

$$\mathbf{W}^{(t)-1}\mathbf{S} = \mathbf{W}^{b(t)-1}\mathbf{S} - \mathbf{W}^{b(t)-1}\mathbf{U}_r \left(\Lambda_r^{-1} + \mathbf{U}_r \mathbf{W}^{b(t)-1}\mathbf{U}_r\right)^{-1} \mathbf{U}_r^\top \mathbf{W}^{b(t)-1}\mathbf{S}, \tag{26}$$

where $\mathbf{W}^{b(t)} = \frac{\sigma_e^{2(t)}}{\sigma_g^{2(t)}}\mathbf{I} + \mathbf{R}^b$. We can easily compute the first term in Supplementary Equation (26) by taking advantage of its banded structure. We calculate the second term of Supplementary Equation (26) from right to left, keeping the order of matrix-vectir multiplication to $\mathscr{O}(pr^2)$ instead of $\mathscr{O}(p^3)$. Since $\left(\Lambda_r^{-1} + \mathbf{U}_r \mathbf{W}^{b(t)-1}\mathbf{U}_r\right)$ is low-rank, its inversion only costs $\mathscr{O}(r^3)$. Overall, we bring down the cost of computing $\mathbf{W}^{(t)-1}\mathbf{S}$ from $\mathscr{O}(p^3)$ to $\mathscr{O}(pr^3 + bp^2)$.

To compute $tr(\mathbf{W}^{(t)-1})$, we take the trace on both sides of the Woodbury identity for $\mathbf{W}^{(t)}$,

$$\begin{aligned} tr(\mathbf{W}^{(t)-1}) &= tr(\mathbf{W}^{b(t)-1}) - tr\left(\left(\mathbf{W}^{b(t)-1}\mathbf{U}_r v_r\right)^\top \left(\mathbf{W}^{b(t)-1}\mathbf{U}_r v_r\right)\right) \\ &= tr(\mathbf{W}^{b(t)-1}) - \left|\left|\mathbf{W}^{b(t)-1}\mathbf{U}_r v_r\right|\right|_F^2, \end{aligned} \tag{27}$$

where $v_r = \left(\Lambda_r^{-1} + \mathbf{U_r}^\top \mathbf{W}^{b(t)-1}\mathbf{U_r}\right)^{-\frac{1}{2}}$ and we re-write the trace-inverse term using matrix form. Since the quantity $\mathbf{W}^{b(t)-1}$ has been calculated in the former step Supplementary Equation (26), both of the two terms on the right-hand side can be easily obtained. Hence the cost of computing $tr(\mathbf{W}^{(t)-1})$ is reduced from $\mathscr{O}(p^3)$ to $\mathscr{O}(bp^2)$. We also avoid storing the full p^2 matrix from iteration to iteration, so that the memory cost of our algorithm is kept at $\mathscr{O}(p\min\{b,r\})$.

Supplementary Note 11

An important aspect of our low-dimensional LD representation algorithm is the tuning of the hyper-parameters. While heuristics or prior knowledge about the structure of the LD can be used to determine the optimal values of (b,r), we used a more principled way to evaluate the performance of a representation. We propose using a data-adaptive procedure to identify the best representation of the LD matrix, using synthetic phenotypic data and cross-validation (Algorithm 1). We also propose an incremental SVD

algorithm to tune the hyperparameters more efficiently, where the number of low-rank factors increases step by step and the performance of the HEELS estimator is evaluated dynamically (Algorithm 2). The incremental SVD algorithm is applicable to "Seq_Band_LR" and "PSD_Band_LR" strategies.

Algorithm 1: Pseudocode for selecting hyperparamter values of b, r

Data: $\mathbf{X} \in \mathbb{R}^{n \times p}$
Input : Strategy (one of the strategies in Table 2),
h^2 (arbitrary value between 0 and 1, used for generating synthetic phenotypes),
m (number of replicates for cross-validation),
$\text{tol}_b, \text{tol}_r$ (stopping criteria, $tol_b < tol_r$),
s (step size, default value is 50),
λ (minimum sparsity, default value is 0.1)
Initialize : $r = 0, b = 0, e_{CV} = 1$

```
while e_CV > tol_b & b < λp do
    b ⟵ b + s;
    while e_CV > tol_r & r < λp do
        r ⟵ r + s;
        (R̂_b, Û_r, Λ̂_r) ⟵ HEELS_sparse_LD(R, b, r, Strategy)
        for i = 1, 2, ..., m do
            β_i ~ N(0, h²/p);   y_i = Xβ_i;
            ĥ²_i ⟵ HEELS_estimate_h²(X^⊤ y_i, R̂_b, Û_r, Λ̂_r)
        end
        e_CV = |(1/m) Σ_{i=1}^{m} ĥ²_i − h²|; ;          /* Cross-validation error */
    end
end
b*, r* ⟵ b, r;
R*_b, U*_r, Λ*_r ⟵ R̂_b, Û_r, Λ̂_r
```

Output : b^*, r^* (optimal hyperparameter values),
$\mathbf{R}_b^*, \mathbf{U}_r^*, \Lambda_r^*$ (optimal representation)

To speed up the low-rank decomposition, we replaced the *exact* solutions based on direct eigen-decomposition with the *approximate* solutions, and compared two approaches – one is the optimization approach ("optim"), where we solve $\arg\min_{\mathbf{U}^r \in L_p(\mathbb{R})} ||\mathbf{R}_{resid} - \mathbf{U}^{r\top}\mathbf{U}^r||_F^2$, and $\mathbf{R}_{resid}$ is the residual off-banded component to be decomposed or approximated and $L_p(\mathbb{R})$ is the collection of $p \times p$ lower triangular matrices; the other approach is based on random sketching ("random"), where we decompose $\mathbf{R}_{resid}$ via SVD[36]. The simulation results indicate that using the optimization approach leads to better approximation of the LD and less variable h^2_{SNP} estimates (Supplementary Figure 14). While we observe larger bias when the randomized SVD approach is used to approximate the low-rank component, it can be more advantageous in applications with larger problem size.

Algorithm 2: Incremental SVD algorithm for selecting optimal r

```
Data: R* = R − R̂_b (residual after the banded component has been estimated)
Input     : tol (stopping criteria), s (increment size), λ (minimum sparsity)
Initialize : r = 0, i = 0, R_r^0 = 0
while e > tol & r < λp do
    i ⟵ i + 1;
    r ⟵ r + s ;
    (Û_r^i.Λ̂_r^i) ⟵ arg min_{Λ_r,U_r} ||R_* − R_r^{i−1} − U_r Λ_r U_r^⊤||_F^2;
    R_r^i ⟵ R_r^{i−1} + Û_r^i Λ̂_r^i Û_r^{i⊤};
    e ⟵ ||R* − R_r^{i−1}||_F / ||R*||_F ;    /* Approximation error of the current low-rank
      representation */
end
r* ⟵ r; U_r^* ⟵ ∑_i U_r^i; Λ_r^* ⟵ ∑_i Λ̂_r^i
Output    : r* (optimal hyperparameter value), U_r^*, Λ_r^* (optimal low-rank representation)
```

Supplementary Figures

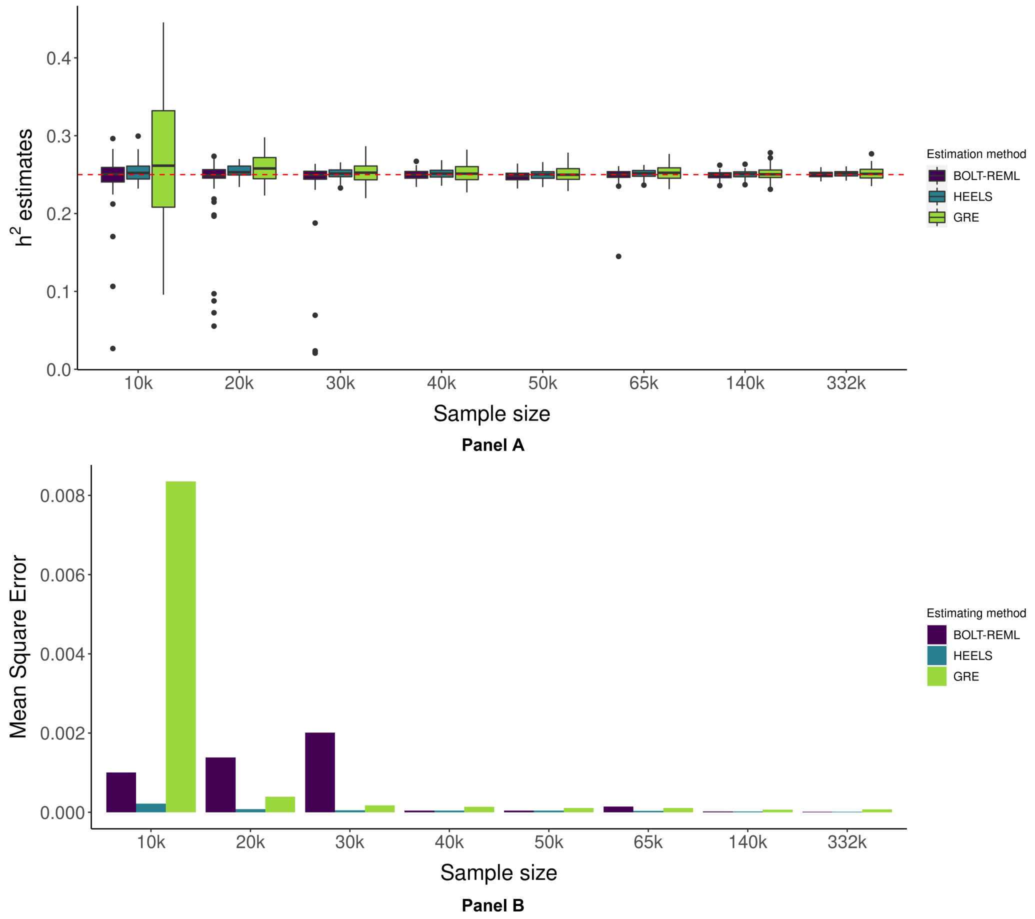

Supplementary Figure 1. Comparison of the performance of $\hat{h}^2_{SNP}$ from GRE and HEELS with various sample sizes. Simulation results based on real genotypic data from random subsets of unrelated individuals in the UK Biobank, array SNPs on chromosome 22 with MAF > 0.01. **Panel A**: Distribution of the h^2_{SNP} estimates. Red-dotted line: true h^2 of 0.25. **Panel B**: MSE of the h^2_{SNP} estimates for each method. Estimates from GREML are omitted as it is not recommended for use when sample size exceeds 30k.

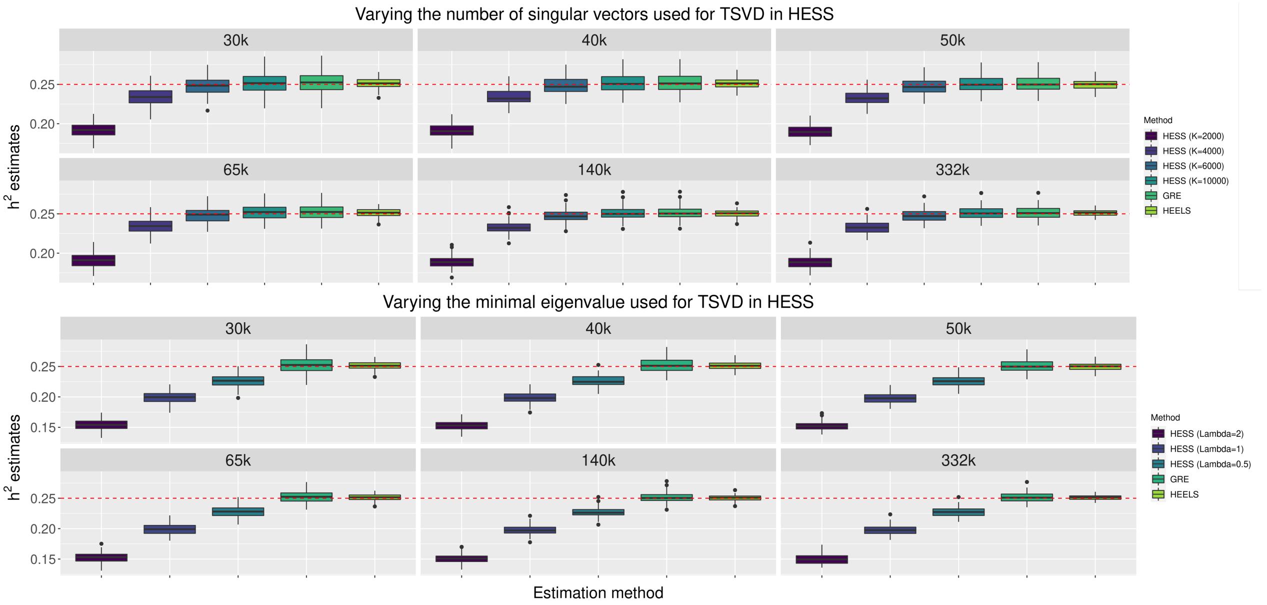

Supplementary Figure 2. Comparison of h^2_{SNP} estimates between HESS, GRE and HEELS with varying levels of regulation in HESS. Different settings of LD regularization are used for HESS. Top panel: Varying number of top eigenvectors (K) used for regularization of LD in HESS. The lower and upper hinges correspond to the first and third quartiles (the 25th and 75th percentiles). The upper (lower) whisker extends from the hinge to the largest (smallest) value no further than $1.5 \times IQR$ from the hinge (IQR: inter-quartile range). Data beyond the end of the whiskers are called "outlying" points and are plotted individually. Bottom panel: Varying minimum eigenvalue (λ) used to regularize the LD. The h^2_{SNP} estimates from GRE and HEELS are identical in the top and bottom panel, included for references. Sample size varies between sub-panels.

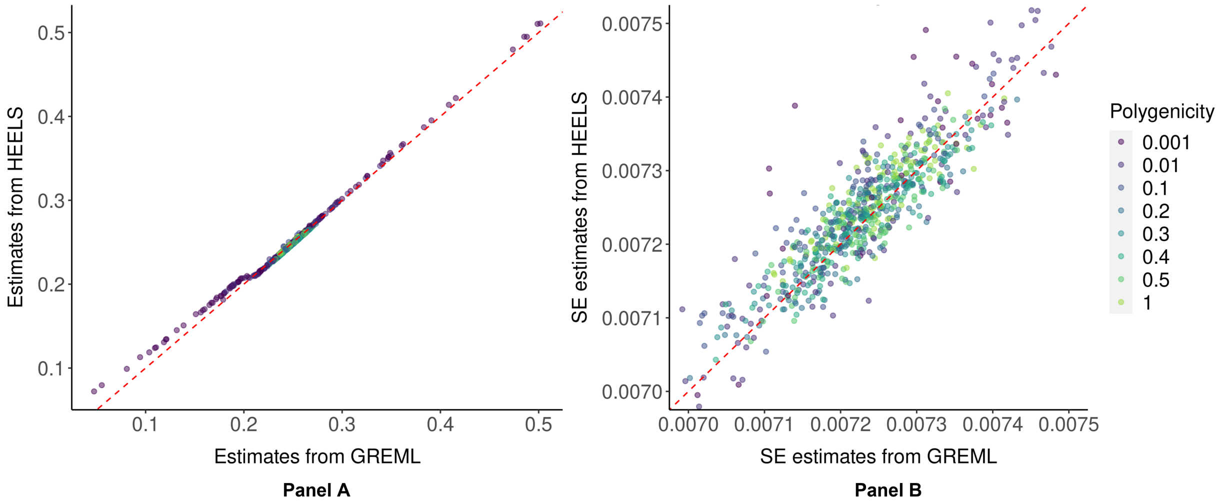

Supplementary Figure 3. High degree of concordance between HEELS and GREML across different polygenicity levels using simulation studies. Simulation results using the real genotypic data of a random sub-sample of individuals from the UK Biobank ($n = 30,000, p = 9,205$). **Panel A**: h^2_{SNP} estimates from HEELS vs GREML. Red-dotted line: $y = x$. **Panel B**: analytical SE estimates from HEELS vs GREML. Polygenicity: proportion of the markers that are causal in the simulation.

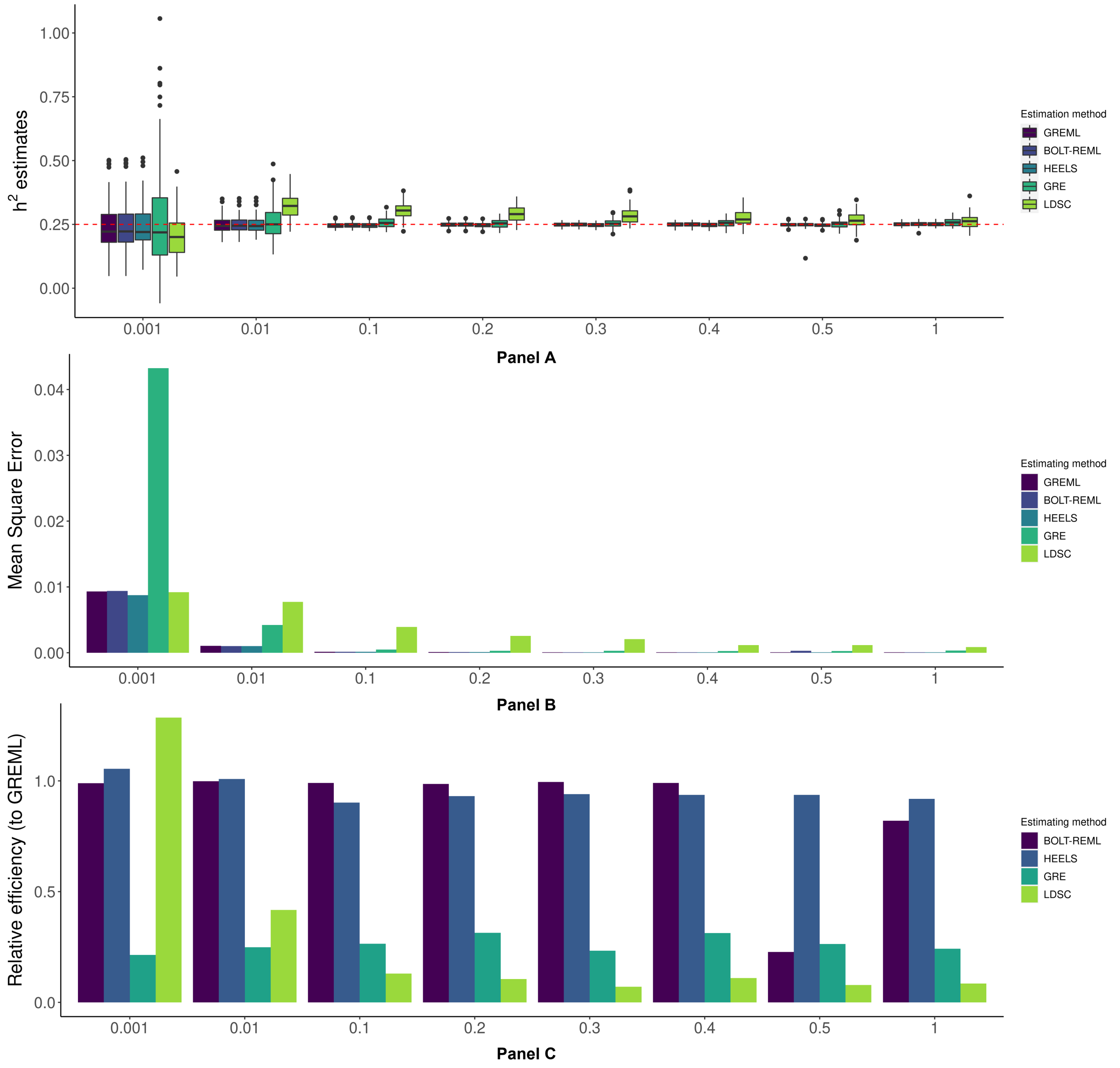

H

Supplementary Figure 4. Comparison of the performance of $\hat{h}^2_{SNP}$ across different methods at various polygenicity levels. Simulation results based on real genotypic data of a random sub-sample of individuals from the UK Biobank ($n = 30,000, p = 9,205$). X-axis represents polygenicity or the proportion of markers that are causal. **Panel A**: Distribution of the h^2_{SNP} estimates from different methods. Red-dotted line: true h^2 of 0.25. **Panel B**: MSE of the h^2_{SNP} estimates for each method. **Panel C**: Relative efficiency of each method compared to GREML. GREML estimates are computed using GCTA; BOLT-REML estimates are computed using the BOLT software.

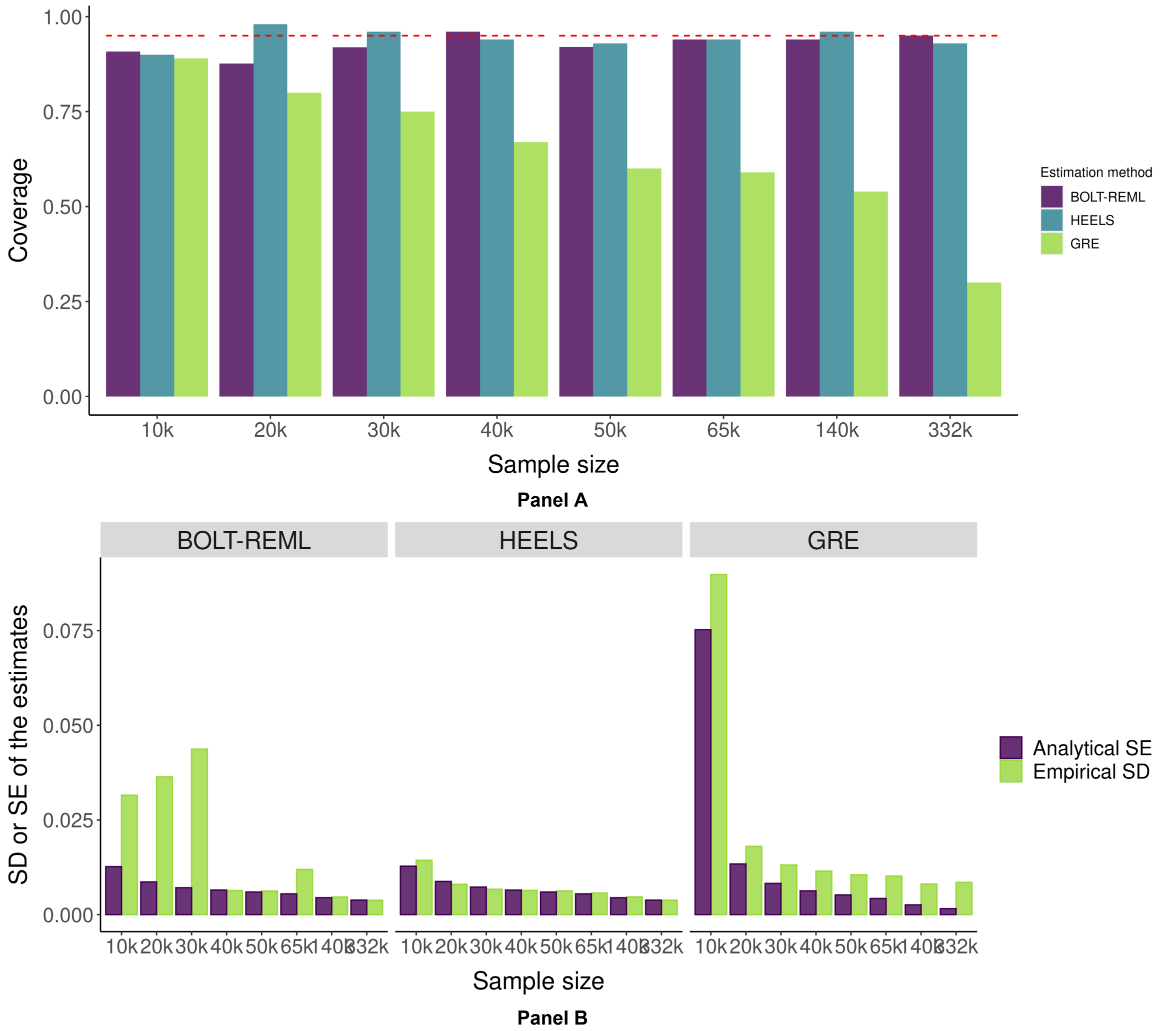

Supplementary Figure 5. Comparison of SE calibration between GRE and HEELS with various sample sizes. Simulation results based on real genotypic data from random subsets of unrelated individuals in the UK Biobank, array SNPs on chromosome 22 with MAF > 0.01. **Panel A**: Proportion of the confidence intervals (constructed using theoretical standard error) that cover the true h^2 value. Red-dotted reference line: 95%, correct coverage if the theoretical standard error is well-calibrated. **Panel B**: Empirical SD of the heritability estimates across experiments or average of the analytical SE reported by different methods.

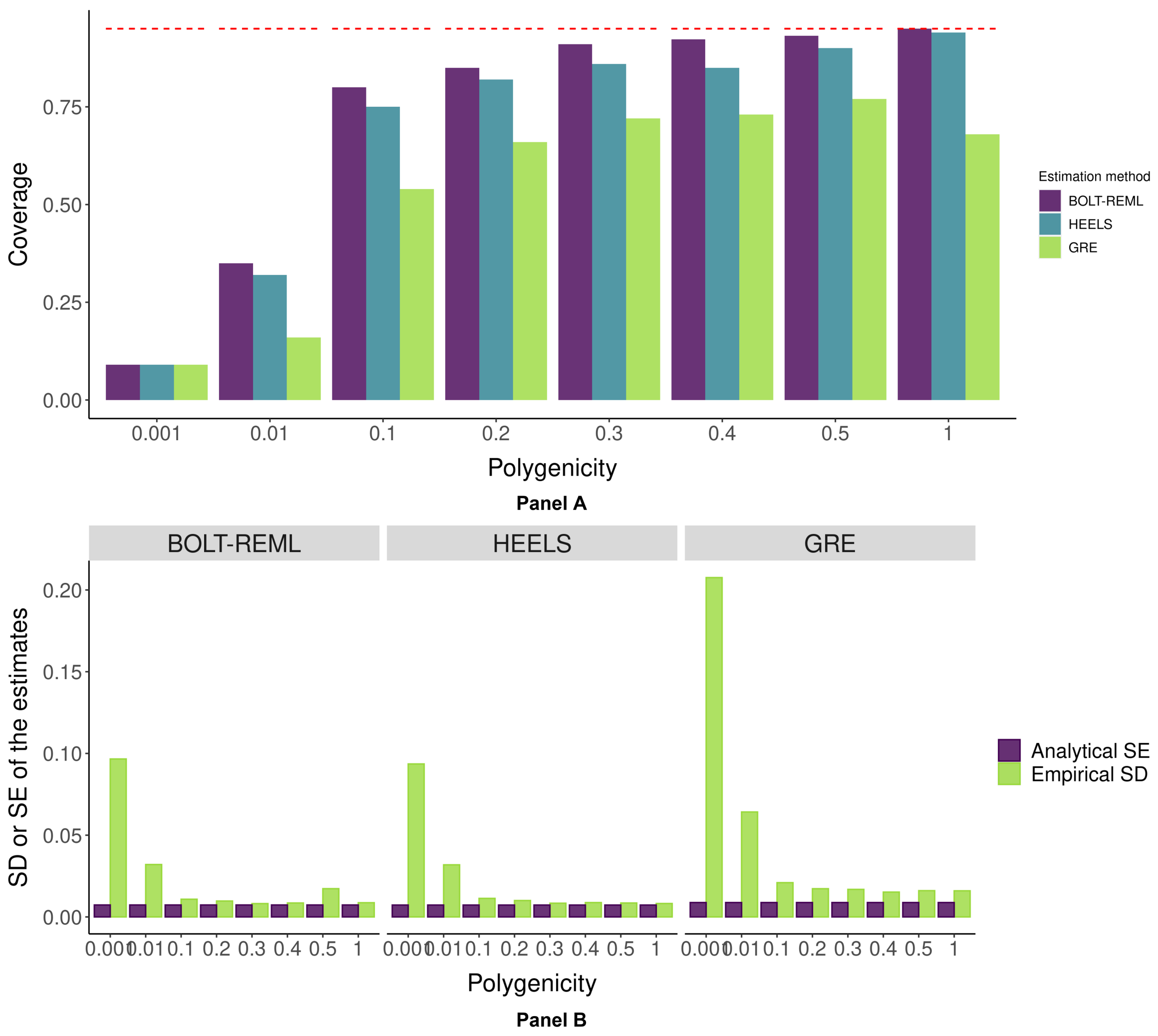

Supplementary Figure 6. Comparison of SE calibration between GRE and HEELS with various levels of polygenicity. Simulation results based on real genotypic data from a random subset of 30,000 unrelated individuals in the UK Biobank, array SNPs on chromosome 22 with MAF > 0.01. **Panel A**: Proportion of the confidence intervals (constructed using theoretical standard error) that cover the true h^2 value. Red-dotted reference line: 95%, correct coverage if the theoretical standard error is well-calibrated. **Panel B**: Empirical SD of the heritability estimates across experiments or average of the analytical SE reported by different methods.

Supplementary Figure 7-14: Unless otherwise specified, all simulation results related to the comparison of LD approximation strategies are based on the simulated phenotypes using real genotypic array data from full UK Biobank on chromosome 22 ($n = 332,430, p = 9,220$).

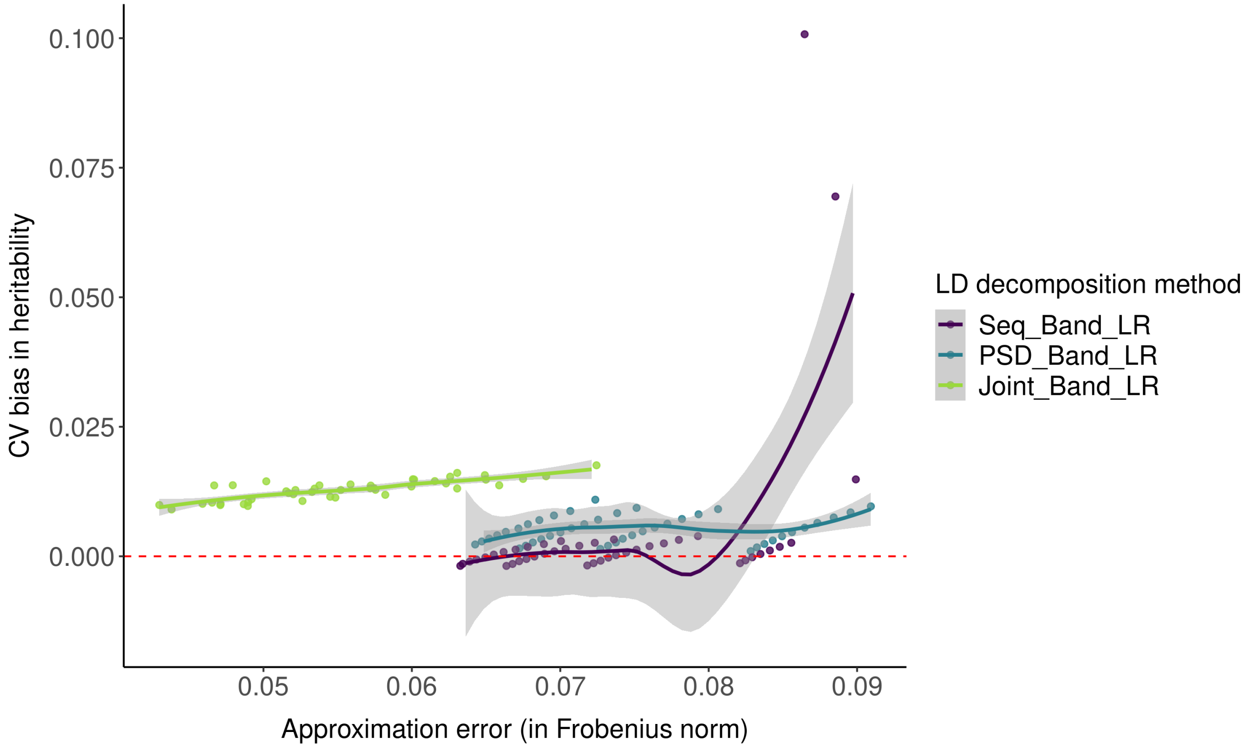

Supplementary Figure 7. Cross-validation (CV) bias of h^2_{SNP} vs LD approximation error of different Banded + LR approximation strategies. Each dot represents one approximation strategy, characterized both by the structure of the LD low-dimensional representation approximation strategy and by the (b, r) values. The same set of hyperparameter values are used for comparison among the three strategies. b varies from 300 to 600 in increments of 100. r ranges from 300 to 800 in increments of 50. X-axis signifies $\frac{||\mathbf{R}-\tilde{\mathbf{R}}||_F}{||\mathbf{R}||_F}$. Y-axis signifies the bias from CV based on 100 simulated phenotypes.

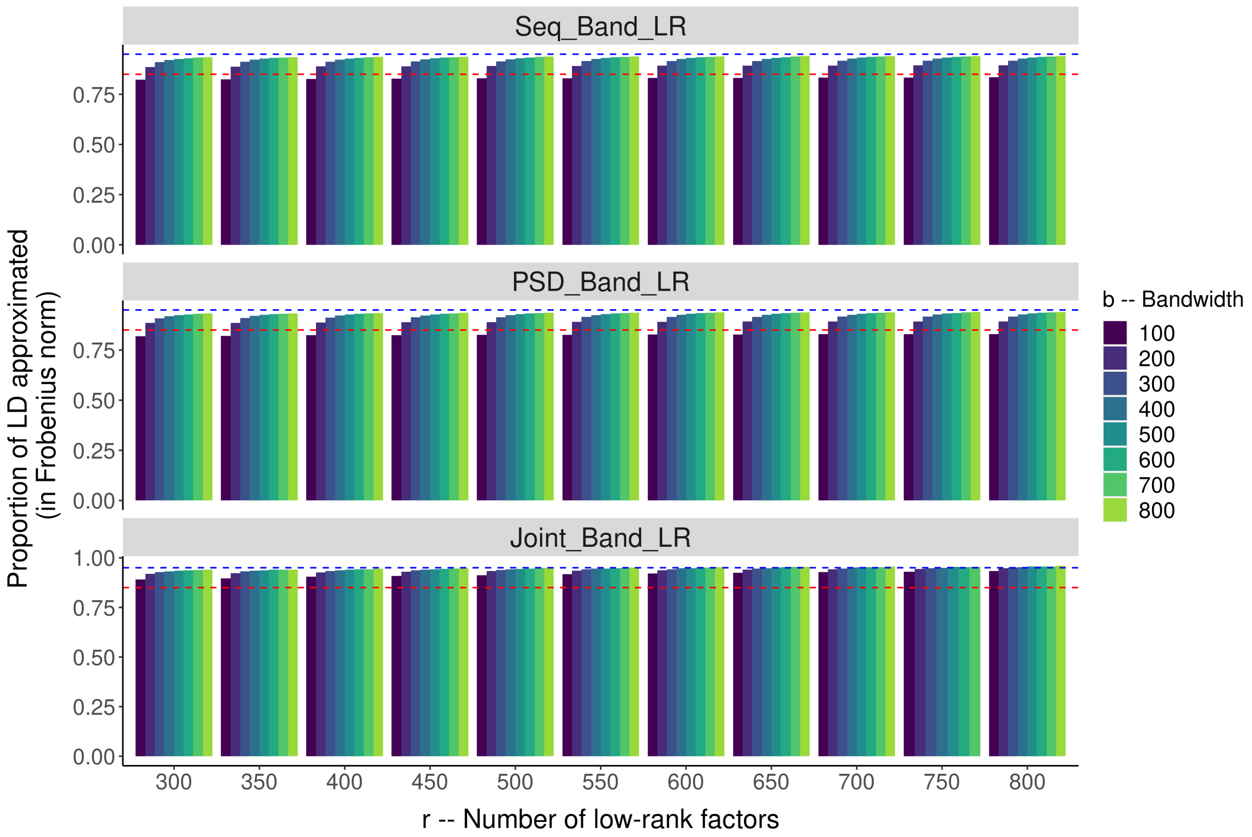

Supplementary Figure 8. Comparison of approximation accuracy between different Banded + LR strategies. Y-axis signifies the approximation accuracy, measured in $\frac{||\mathbf{R}_b+\mathbf{R}_r||_F}{||\mathbf{R}||_F}$. Each bar represents one specific LD approximation setting, which is specified by both the decomposition strategy and the hyperparameter values (b,r).

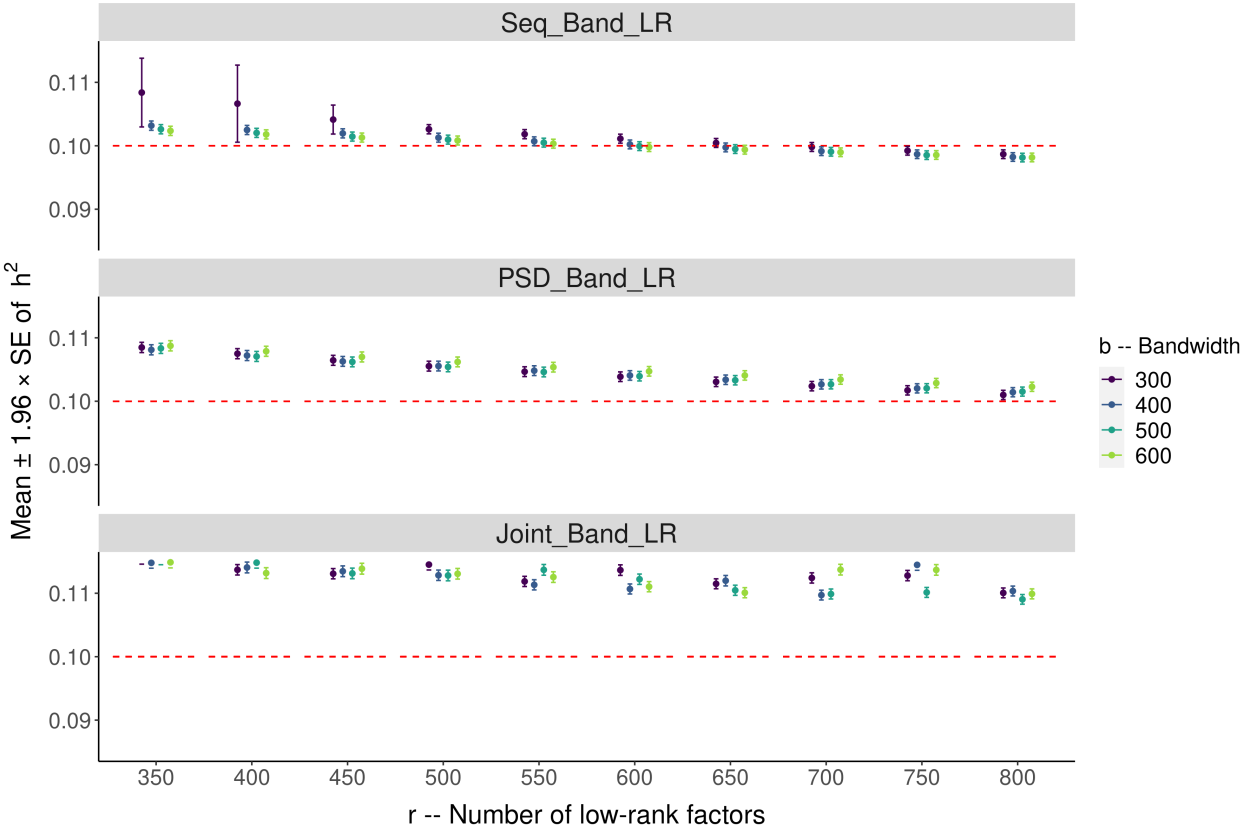

Supplementary Figure 9. Comparison of h^2_{SNP} estimates among different Banded + LR approximation strategies. Each point and bar represents one specific LD approximation setting. The points correspond to the average h^2 estimates across 100 simulations, and the upper (lower) whisker extends from the mean to the values $1.96 \times SE$ above (below) the mean. Red-dotted reference line: true heritability of 0.1.

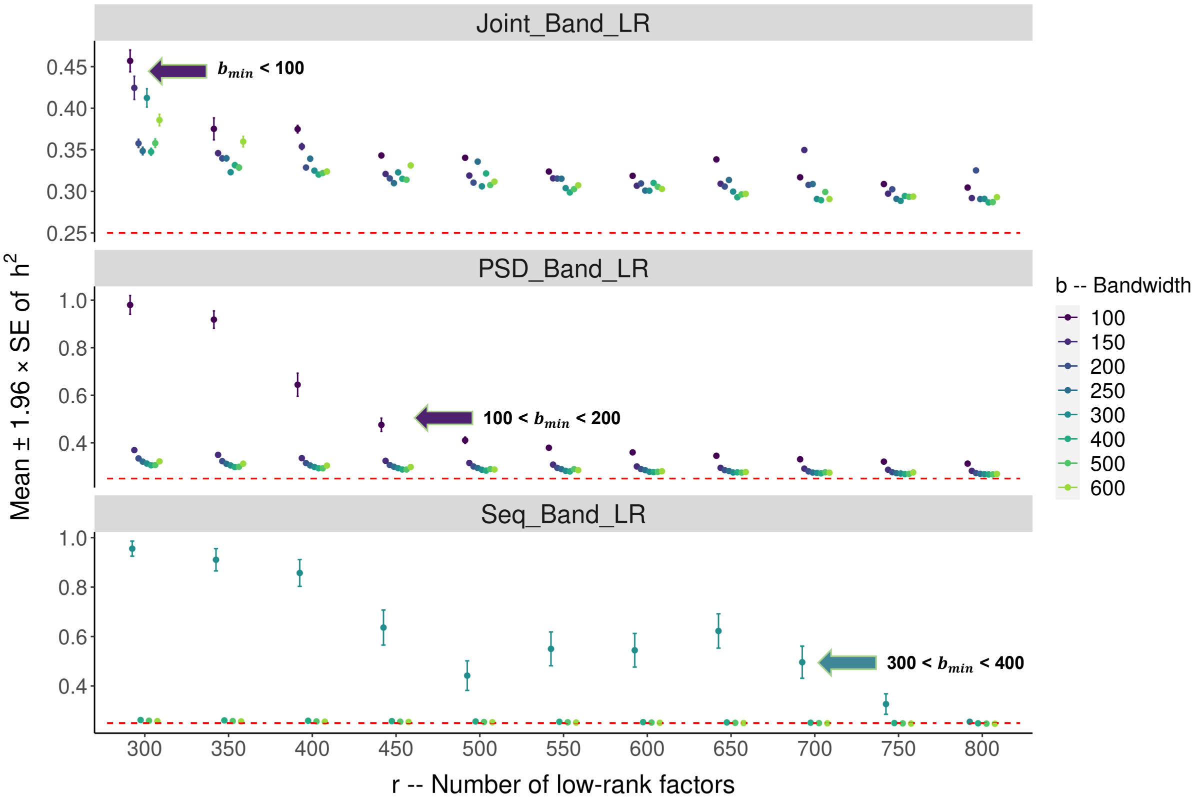

Supplementary Figure 10. The bandwidth of $\widetilde{\mathbf{R}}_b$ critically determines the bias of h^2_{SNP} estimates from HEELS. b_{min} signifies the threshold value of the bandwidth which heavily influences the bias of h^2_{SNP}, *i.e.* widening the bandwidth up to this value can substantively reduce the bias in h^2_{HEELS}, but such "debiasing" effect diminishes after b exceeds this threshold b_{min}. Each point and bar represents one specific LD approximation setting. The points correspond to the average h^2 estimates across 100 simulations, and the upper (lower) whisker extends from the mean to the values $1.96 \times SE$ above (below) the mean. Red-dotted reference line: true heritability of 0.25.

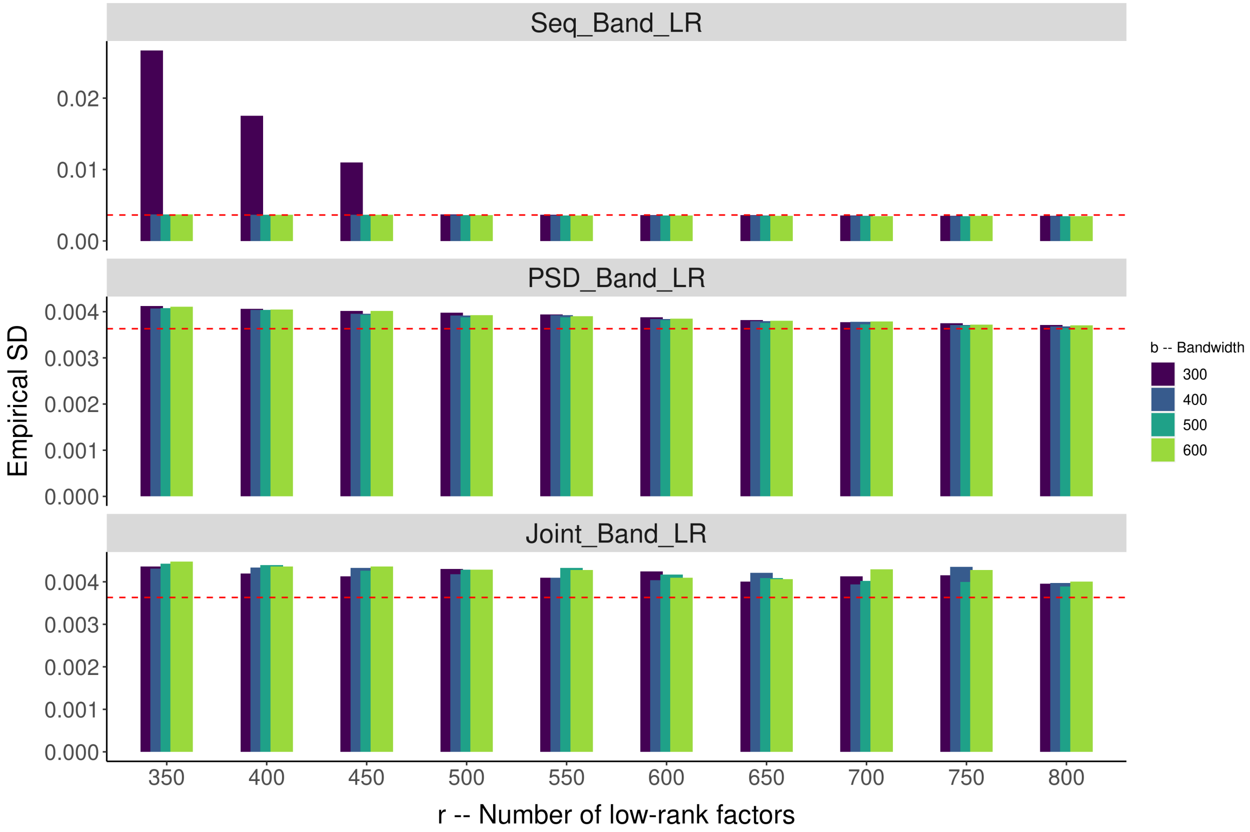

Supplementary Figure 11. Comparison of the empirical SD of h^2_{SNP} estimates among different Banded + LR approximation strategies. Each bar represents the empirical SD of the h^2_{SNP} estimates across 100 simulations, based on one specific LD approximation setting. Red-dotted reference line: empirical SD of the h^2 estimates when the full LD is used.

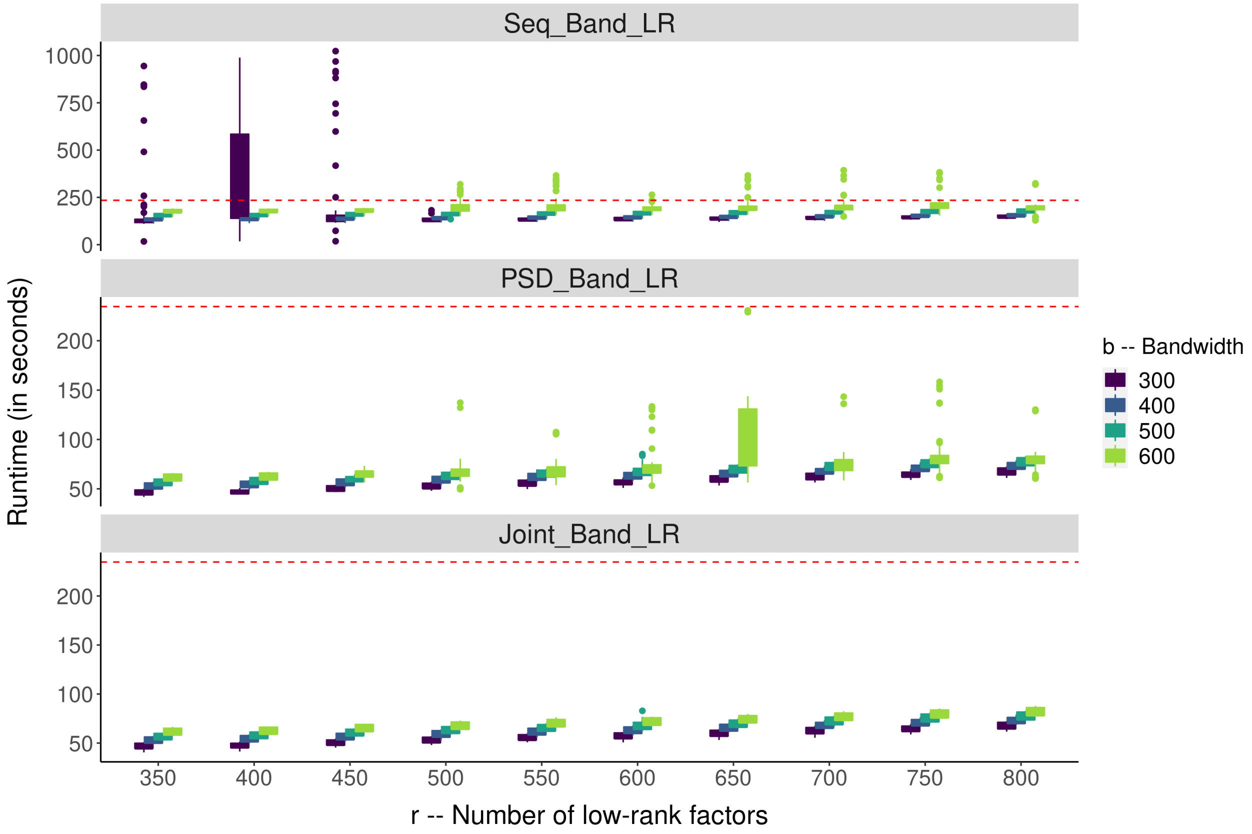

Supplementary Figure 12. Runtime comparison among different Banded + LR approximation strategies. Each boxplot represents the distribution of runtime across 100 simulations. Runtime is closely related to the cost of inverting the approximating matrix. It is evident from this figure that two factors can result in longer runtime: non-PSD of the approximating matrix, which is the case for certain "Seq_Band_LR" strategies due to the lack of PSD guarantee and larger bandwidth of the banded component, which notably increases the runtime of all Banded + LR strategies. Red dotted reference line: average runtime from using the exact full LD.

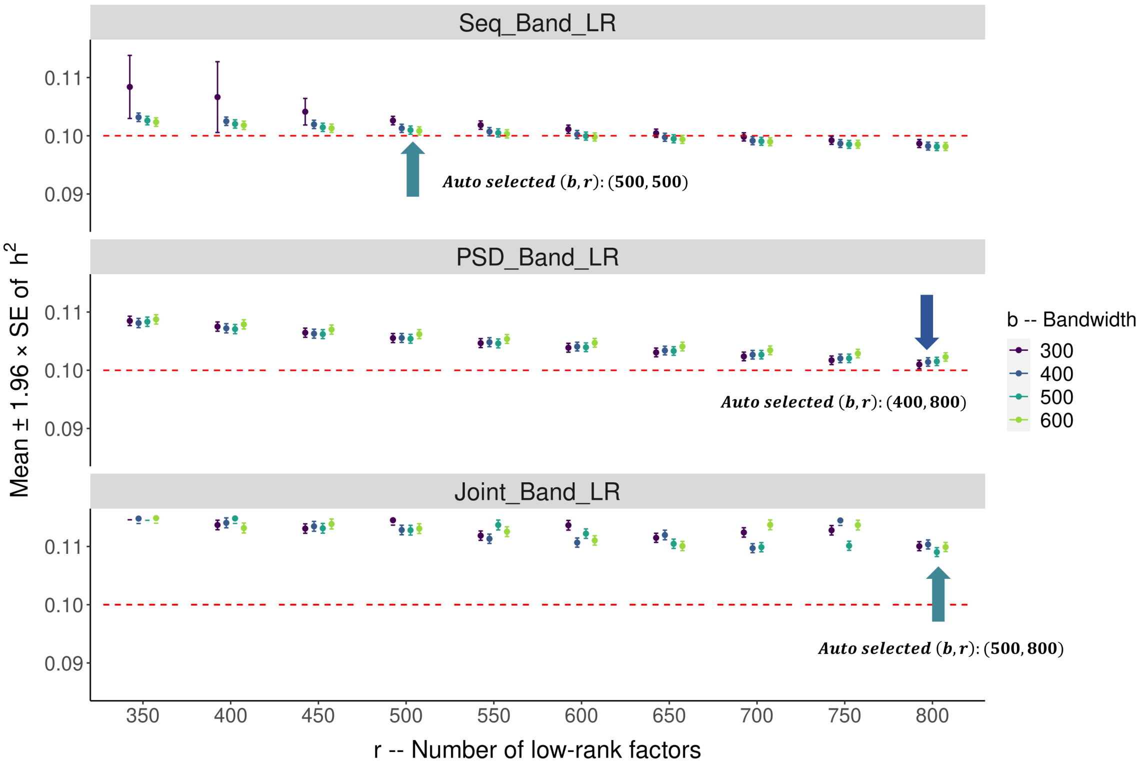

Supplementary Figure 13. Hyperparameter selection for the Banded + LR strategies from pseudo-validation. Arrows point to the best-performing hyperparameter settings validated from an independently simulated set of phenotypes. The upper (lower) whisker extends from the mean to the values $1.96 \times SE$ above (below) the mean. Due to the stochastic nature of the simulated phenotypes, the selected pairs of (b, r) do not necessarily lead to the lowest bias in h^2_{SNP} when tested on a *new* set of simulated phenotypes, but we note that the selected settings lead to comparably small degree of bias.

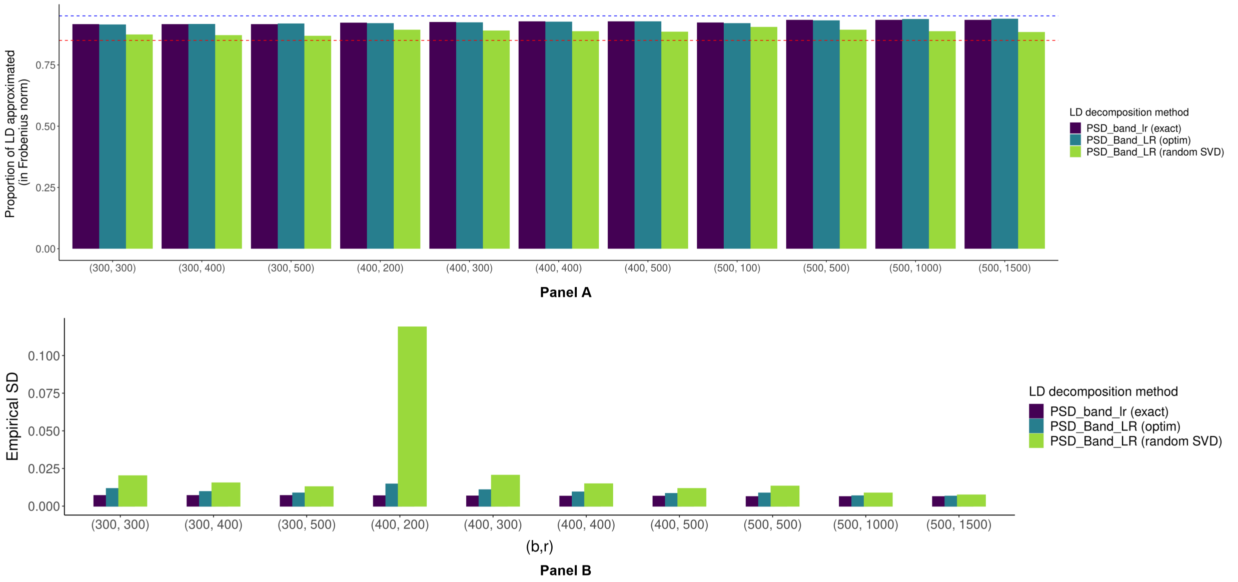

Supplementary Figure 14. Comparing the performance of *exact* vs *approximate* solutions to low-rank decomposition. **Panel A**: Approximation accuracy, measured by $||\tilde{\mathbf{R}}||_F/||\mathbf{R}||_F$, where $\tilde{\mathbf{R}}$ is the LD approximation. Dotted lines are the reference levels: red – 85%; green – 95%. **Panel B**: Empirical SD of heritability estimates. PSD_Band_LR (optim) uses optimization to calculate the low-rank component; PSD_Band_LR (random SVD) uses the sketching method to compute the low-rank component fast. b: bandwidth of the banded component. r: number of factors in the low-rank component.

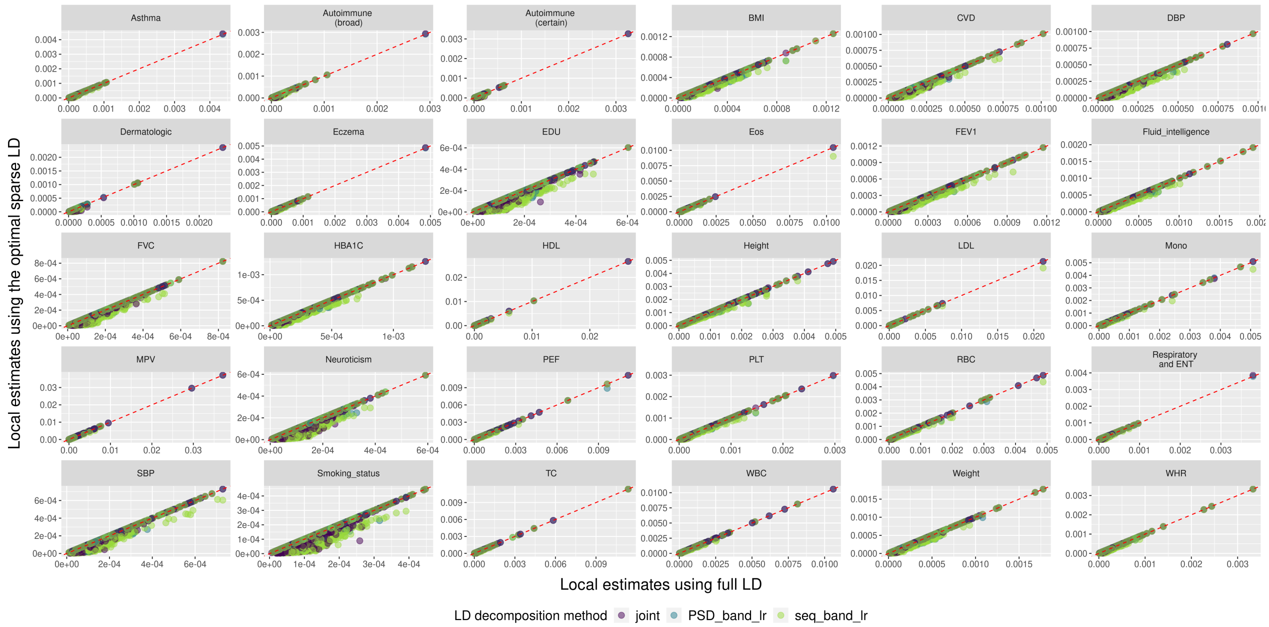

Supplementary Figure 15. Comparison of local heritability estimates between using full LD and using sparse representations. Red dotted reference line: $y = x$.

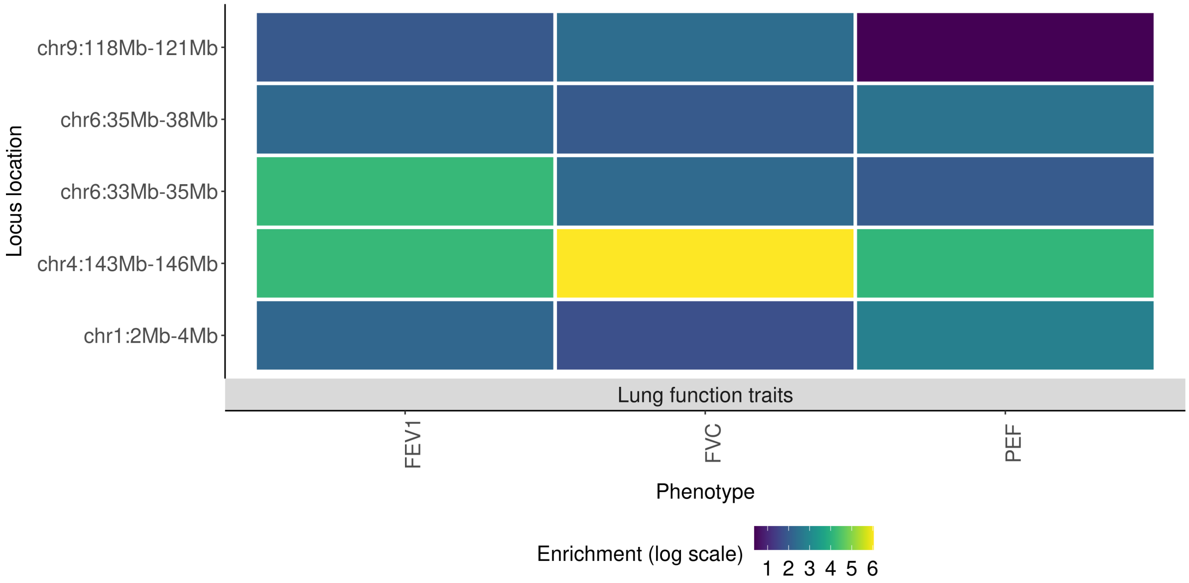

Supplementary Figure 16. Prioritized pleiotropic loci for lung function traits using the UKB data. The plotted regions host significant local heritability for at least two pulmonary function traits.

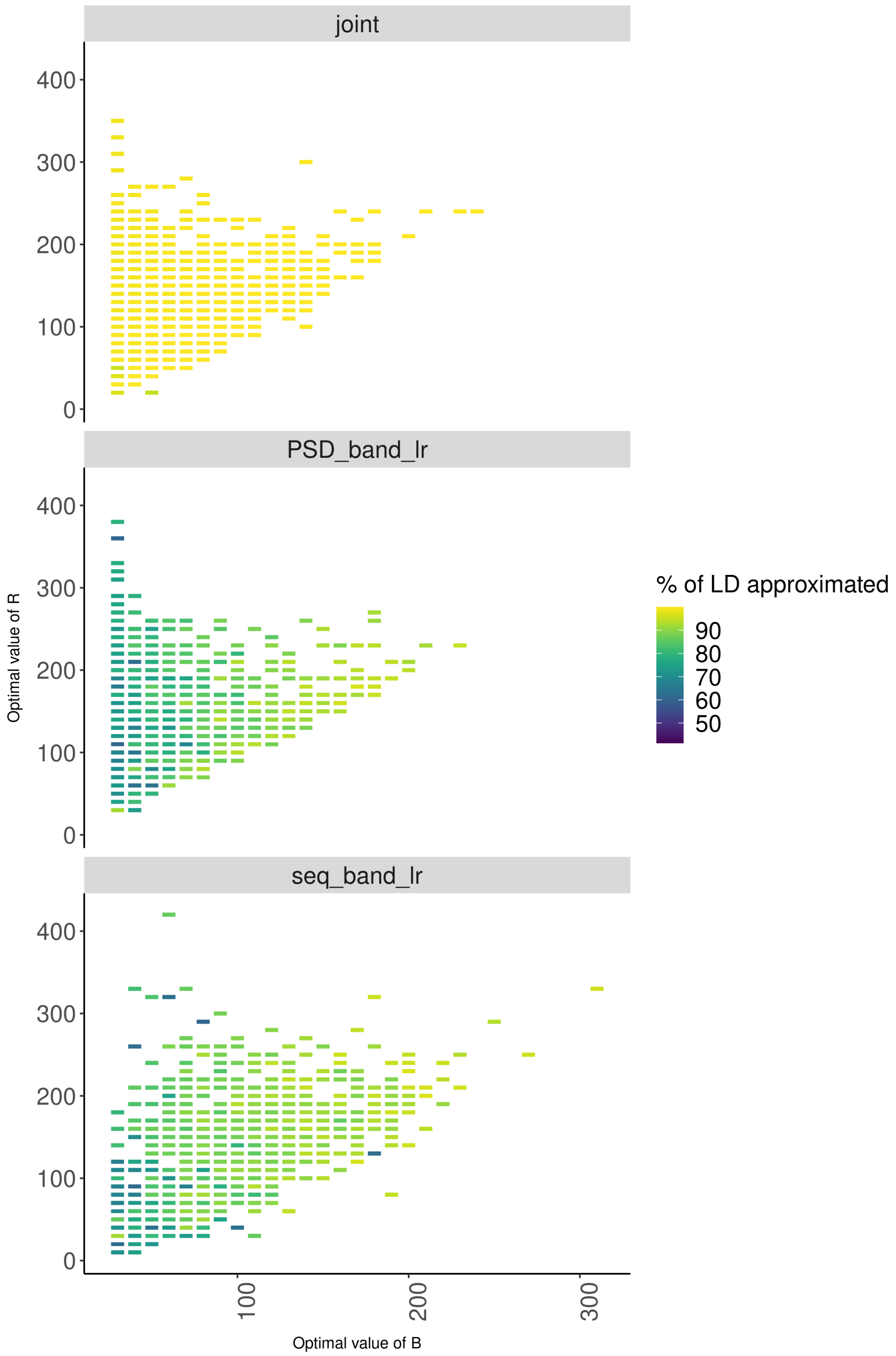

Supplementary Figure 17. Comparison of LD approximation accuracy across different strategies. Axes represent the optimally selected hyperparamter values. The shade of the tiles represents the extent of LD approximated. Lighter color corresponds to better approximation.

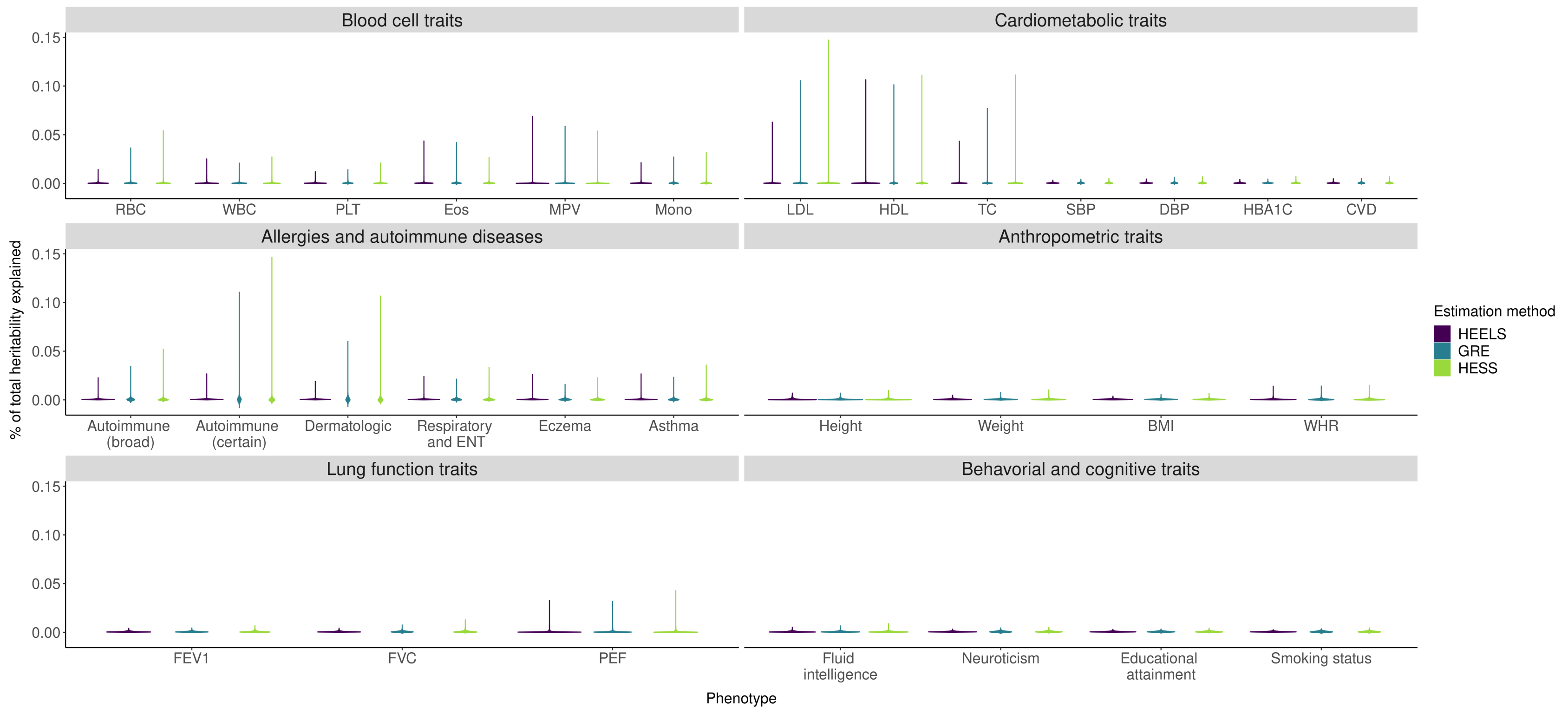

Supplementary Figure 18. Distribution of local heritability estimates in the UKB empirical meta-analysis ($n = 332,340$). Traits are grouped based on their phenotype categories.

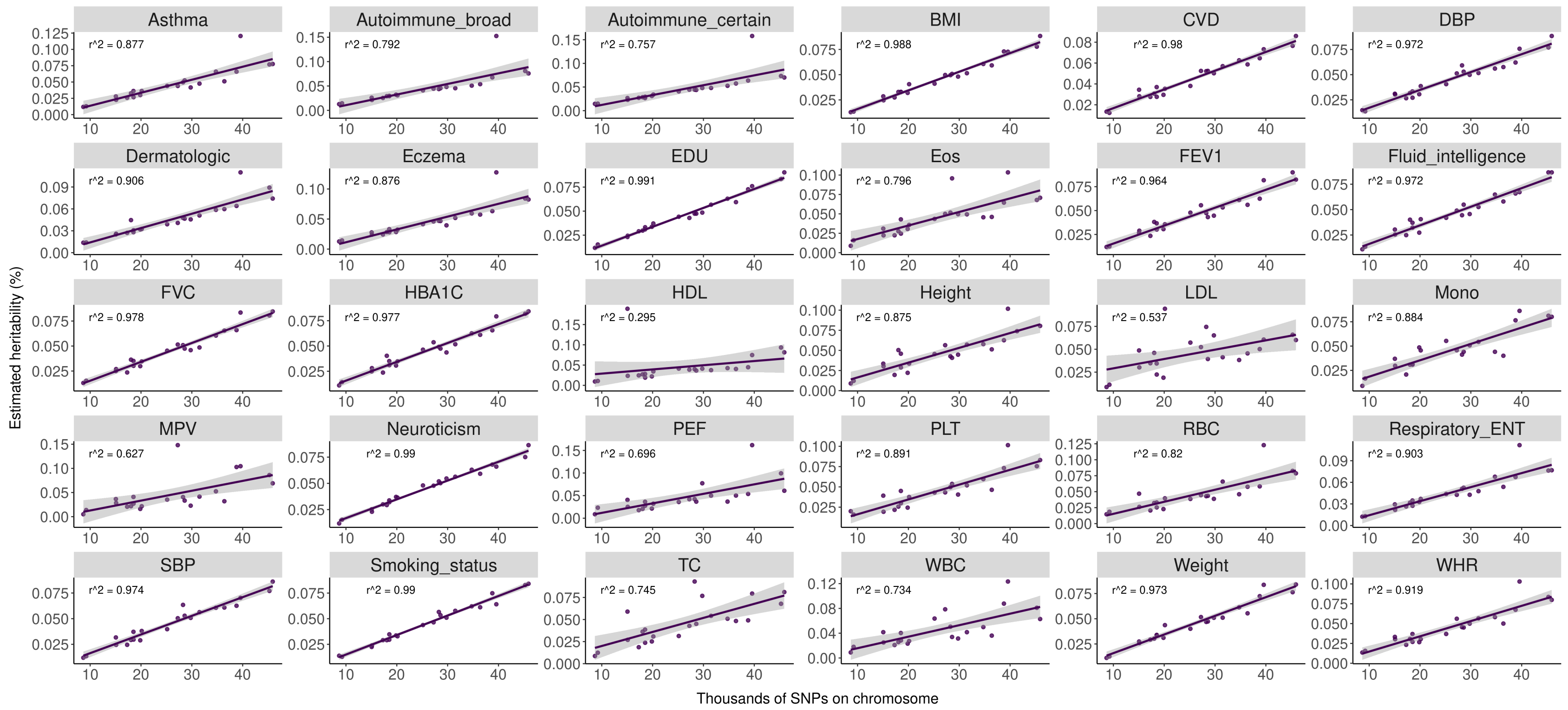

Supplementary Figure 19. Relationship between length and the proportion of h^2_{SNP} contributed by different chromosomes in the UKB empirical meta-analysis ($n = 332,340$). Length of the chromosome is measured by the number of SNPs.

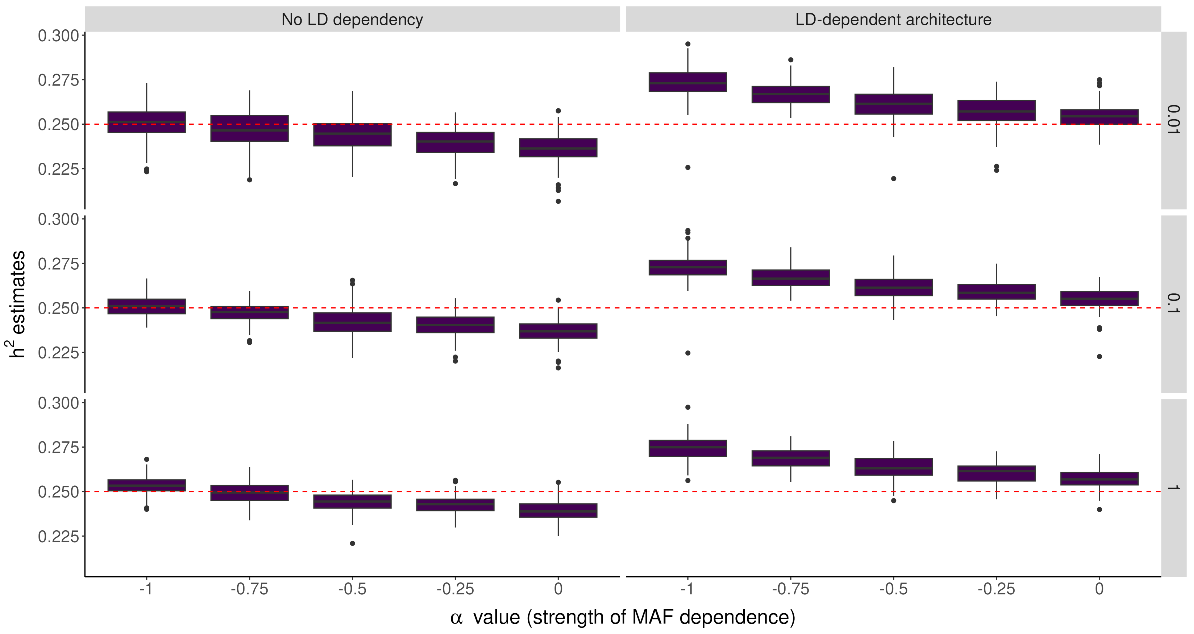

Supplementary Figure 20. Distribution of $\hat{h}^2_{SNP}$ estimates from HEELS under mis-specified models (MAF and LD-dependent). Simulation results based on real genotypic data from random subsets of unrelated individuals in the UK Biobank, array SNPs on chromosome 22 with MAF > 0.01. The value of α determines the strength of the MAF-dependency[37], $\sigma_j^2 \propto (f_j(1-f_j))^{1+\alpha}$. Setting α to -1 corresponds to the GCTA model; setting α to -0.25 corresponds to the LDAK model[3]. LD-dependent architecture: assume the genetic variance of a variant is inversely proportional to its level of LD. Red-dotted line: true heritability of 0.25. The row panels represent polygenicity or the proportion of SNPs with non-zero effects, e.g., 0.1 means that 10% of the SNPs are causal.

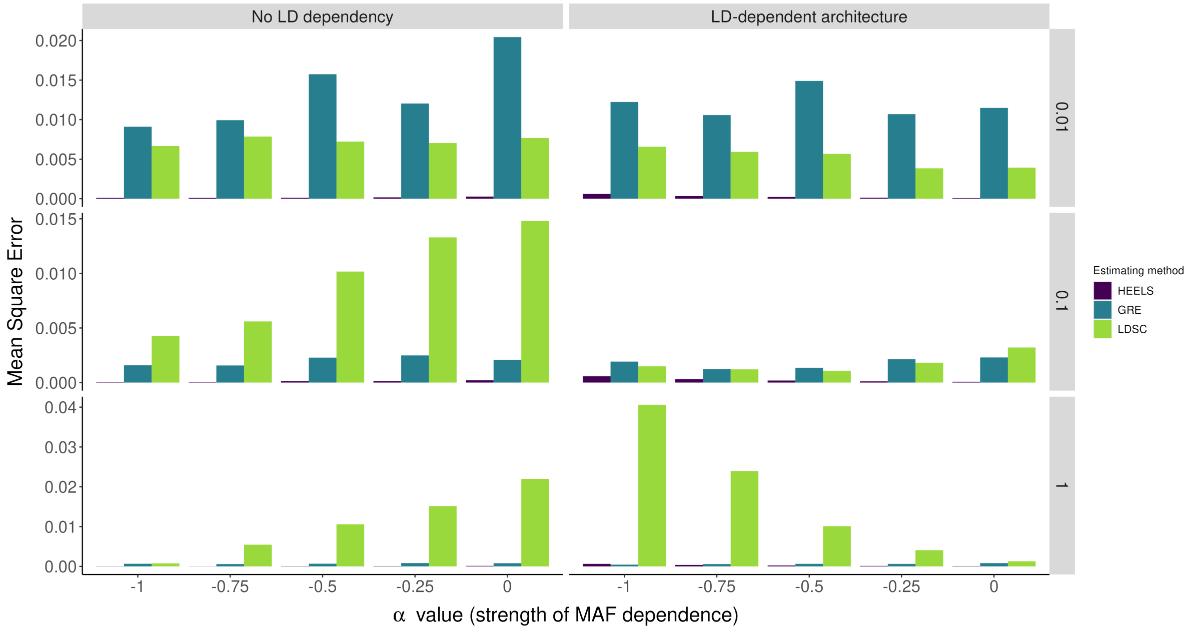

Supplementary Figure 21. Comparison of MSE between different summary-statistics-based h^2_{SNP} estimation methods under mis-specified models. Simulation results based on real genotypic data from random subsets of unrelated individuals in the UK Biobank, array SNPs on chromosome 22 with MAF > 0.01. The value of α determines the strength of the MAF-dependency[37], $\sigma_j^2 \propto (f_j(1-f_j))^{1+\alpha}$. Setting α to -1 corresponds to the GCTA model; setting α to -0.25 corresponds to the LDAK model[3]. LD-dependent architecture: assume the genetic variance of a variant is inversely proportional to its level of LD. The row panels represent polygenicity or the proportion of SNPs with non-zero effects, e.g., 0.1 means 10% of the SNPs are causal.

Supplementary Tables

Method	Type of input data	Description and assumptions	Computational algorithm	Statistical efficiency
***GREML**	Individual-level	Estimates h^2_{SNP} via REML. Uses the LMM assumptions: sizes are normally distributed with equal variance, independent of MAF or LD.	Maximizes REML using an iterative algorithm. Uses the average information (AI) to approximate the Hessian of log-likelihood. (Other options: Fisher scoring and EM.)	Asymptotic efficiency guaranteed as Cramer-Rao lower bound is attained for REML.
LDAK	Individual-level	Estimates h^2_{SNP} via REML. Assumes normally distributed effect sizes, but allows marker-specific genetic variance to depend on MAF, LD and imputation quality.	Same algorithm as GREML for maximizing REML. Calculating the weighted kinship matrix can be computationally intensive.	Comparable efficiency to GREML[4,20].
BOLT-REML	Individual-level	Same as GREML.	Improves GREML's maximization algorithm using Monte-Carlo sampling for the score and information conjugate gradient iteration to solve the MME, etc. Scalable to biobank-sized dataset.	Slightly less efficient than GREML due to random sampling[2,20].
RHE-reg	Individual-level	Estimates h^2_{SNP} using the HE-regression. Assumes an infinitesimal model but relaxes the distributional assumptions and only uses the second moments.	Improves the original HE-regression estimation procedure using a randomized estimator of trace and employs the Mailman algorithm for fast matrix-vector multiplication. Scalable to biobank-sized dataset.	Lower efficiency than GREML as a MoM estimator. Randomization introduces more uncertainty[9].
BSLMM	Individual-level	Estimates h^2_{SNP} using a hybrid model that generalizes both LMM and the sparse regression model. Uses a mixture of two normals to model effect sizes, capturing the sparse effects and the polygenic effects.	Bayesian inference based on MCMC sampling from the posterior. Incorporates efficient computational tricks such as projection/whitening to avoid expensive matrix operations in likelihood evaluation.	Comparable efficiency to GREML when the LMM assumption is met[11].
***LDSC**	Summary-level	Estimates h^2_{SNP} based on the relationship between LD-tagging and the expected association test statistics. Uses the same assumptions as RHE-reg.	Estimates SNP heritability using iteratively re-weighted least squares in the regression framework. Computationally efficient.	Lower efficiency than GREML as a MoM estimator[19].
SumHer	Summary-level	Extends LDAK to base on summary statistics. Uses the same assumptions as LDSC but explicitly models the MAF and LD-dependent architecture.	Same as LDSC. Computationally efficient.	Comparable efficiency as LDSC[20].
MQS	Summary-level	Estimates h^2_{SNP} based on the method of moments and minimal norm quadratic unbiased estimation criterion. Generalizes HE-regression such that it can be based on summary statistics. Uses the same assumptions as LDSC.	Uses the sub-sampling strategy which helps reduce computation and memory cost.	More efficient than LDSC but less efficient than GREML[19].
RSS	Summary-level	Extends BSLMM to base on summary statistics. Uses the same assumptions as BSLMM.	Bayesian estimation and inference based on MCMC sampling from the posterior. Uses shrinkage estimator to regularize LD[23].	Less efficient than corresponding Bayesian methods that are based on individual-level data, such as BSLMM[24].
HESS	Summary-level	Estimates local h^2_{SNP} via a closed-form solution, derived by treating causal genetic effects as fixed and assuming genotypes are random. Applies truncated SVD to reduce noise in the LD matrix.	Directly computes the closed-form solution. Local estimates are computed for each region.	Variable depending on the degree of LD regularization[21].
***GRE**	Summary-level	Estimates h^2_{SNP} via a closed-form solution, assuming random genetic effects and allowing SNP-specific variance. Same analytical form as HESS, without LD regularization. Requires $n >> p$.	Directly computes the closed-form solution. Large LD matrices are estimated chunk-wise. Requires direct LD matrix inversion.	Less efficient than GREML, but more efficient than LDSC[20].
HDL	Summary-level	Estimates h^2_{SNP} via MLE, using the marginal likelihood of $\mathbf{Z}$-statistics. Uses the same assumptions as LDSC, but extends it and utilize non-diagonal elements of $Cov(\mathbf{Z})$.	Directly maximizes the likelihood using Newton-Raphson. Regularizes LD using truncated SVD.	Not comparable, since the estimator is likely to be biased[26].

Supplementary Table 1. Summary of existing h^2_{SNP} estimation methods. Methods marked with * are directly compared and benchmarked with HEELS in simulations. For methods that we do not directly compare with HEELS, we cite results on the efficiency comparison between them and GREML/LDSC/GRE based on previous studies and the references therein[4,9,11,19,20].

Structure	Strategy name	Form of Decomposition	Estimation steps
Spiked covariance	Spike_LR	$Diag(\sigma^{,}...\sigma^2) + \mathbf{U_r}\Lambda_r\mathbf{U_r}^\top$	1. Pre-estimate σ^2 using a $(r+1)\times(r+1)$ subsample of $\mathbf{R}$<br>2. Low-rank decompose the residual $\mathbf{R} - \hat{\sigma}^2\mathbf{I}$<br>and approximate it by $\mathbf{U_r}\Lambda_r\mathbf{U_r}^\top$
Spiked covariance	Spike_PSD	$Diag(\sigma^2, ...\sigma^2) + \mathbf{L_r}\mathbf{L_r}^\top$	Solve the optimization problem: $\min_{\sigma^2,\mathbf{L_r}} \|\|\mathbf{R} - \sigma^2\mathbf{I} - \mathbf{L_r}\mathbf{L_r}^\top\|\|_F^2$
Spiked covariance	Spike_PSD_hetero	$Diag(\sigma_1^2, ...\sigma_p^2) + \mathbf{L_r}\mathbf{L_r}^\top$	Solve the optimization problem: $\min_{\sigma_1^2,...\sigma_p^2,\mathbf{L_r}} \|\|\mathbf{R} - Diag(\sigma_1^2, ...\sigma_p^2) - \mathbf{L_r}\mathbf{L_r}^\top\|\|_F^2$
Banded + Low-rank	Seq_Band_LR	$\mathbf{R}_b + \mathbf{U_r}\Lambda_r\mathbf{U_r}^\top$	1. Band $\mathbf{R}$ to obtain $\mathbf{R}_b$<br>2. Low-rank decompose the residual $\mathbf{R} - \mathbf{R}_b$ and approximate it by $\mathbf{U_r}\Lambda_r\mathbf{U_r}^\top$
Banded + Low-rank	PSD_Band_LR	$\mathbf{L_b}\mathbf{L_b}^\top + \mathbf{U_r}\Lambda_r\mathbf{U_r}^\top$	1. Band $\mathbf{R}$ to obtain $\mathbf{R}_b$<br>2. Solve the optimization problem: $\widehat{\mathbf{L}}_b = \arg\min_{\mathbf{L_b}} \|\|\mathbf{R}_b - \mathbf{L_b}\mathbf{L_b}^\top\|\|_F^2$<br>3. Low-rank decompose the residual $\mathbf{R} - \widehat{\mathbf{L}}_b\widehat{\mathbf{L}}_b^\top$, approximating it by $\mathbf{U_r}\Lambda_r\mathbf{U_r}^\top$
Banded + Low-rank	Joint_Band_LR	$\mathbf{L_b}\mathbf{L_b}^\top + \mathbf{L_r}\mathbf{L_r}^\top$	Solve the optimization problem: $\min_{\mathbf{L_b},\mathbf{L_r}} \|\|\mathbf{R} - \mathbf{L_b}\mathbf{L_b}^\top - \mathbf{L_r}\mathbf{L_r}^\top\|\|_F^2$

Supplementary Table 2. Estimation procedures of different strategies for sparse representation of LD. PSD: positive semi-definite assumption. *b*: bandwidth of the banded component; *r*: number of low-rank factors. $\{\mathbf{L}, \mathbf{U}, \mathbf{\Lambda}\}$: Cholesky factor, eigenvector and eigenvalue of the target matrix.

		Correlation of h2 estimates with REML			Correlation of SE estimates with REML			RE to REML		
**Trait**	**Trait category**	**HEELS**	**GRE**	**HESS**	**HEELS**	**GRE**	**HESS**	**HEELS**	**GRE**	**HESS**
**BMI**	**Anthropometric traits**	0.99117	0.90553	0.88761	0.98334	0.80279	0.75637	0.91432	0.67338	0.70336
**Height**	**Anthropometric traits**	0.99882	0.94992	0.88221	0.99433	0.82789	0.79068	0.91876	0.68556	0.71682
**Weight**	**Anthropometric traits**	0.99536	0.92411	0.88076	0.98933	0.81221	0.76778	0.90664	0.68217	0.71286
**WHR**	**Anthropometric traits**	0.98987	0.93289	0.93139	0.98597	0.79116	0.72093	0.87513	0.56037	0.59115
**EDU**	**Behavorial traits**	0.94247	0.76555	0.86757	0.94382	0.72254	0.66315	0.86431	0.55030	0.58861
**Fluid_intelligence**	**Behavorial traits**	0.99505	0.93632	0.89735	0.98922	0.83416	0.78570	0.90994	0.66307	0.68879
**Neuroticism**	**Behavorial traits**	0.92921	0.74901	0.84755	0.94529	0.76242	0.71102	0.89545	0.53155	0.55584
**Smoking_status**	**Behavorial traits**	0.92019	0.73125	0.83675	0.93573	0.73071	0.67037	0.84536	0.52654	0.56106
**Eos**	**Blood traits**	0.99721	0.96158	0.86633	0.99397	0.78269	0.69996	0.88347	0.55417	0.58120
**Mono**	**Blood traits**	0.99570	0.94564	0.90468	0.99005	0.81267	0.73045	0.86420	0.53331	0.56024
**MPV**	**Blood traits**	0.99729	0.91122	0.85619	0.99307	0.79001	0.76280	0.88204	0.57445	0.60050
**PLT**	**Blood traits**	0.99441	0.92189	0.87961	0.99255	0.80036	0.77177	0.86788	0.54983	0.56707
**RBC**	**Blood traits**	0.99781	0.87033	0.79092	0.99543	0.79808	0.74291	0.89096	0.59893	0.61751
**WBC**	**Blood traits**	0.99845	0.93262	0.89028	0.99556	0.79014	0.75723	0.89450	0.60330	0.62802
**DBP**	**Cardiometabolic**	0.97275	0.84017	0.89226	0.97335	0.76824	0.72357	0.89870	0.55797	0.58690
**SBP**	**Cardiometabolic**	0.96878	0.84517	0.88741	0.97328	0.78589	0.73259	0.90246	0.55671	0.58980
**HBA1C**	**Cardiometabolic**	0.99128	0.91232	0.91869	0.98629	0.81695	0.76497	0.91065	0.60830	0.65306
**HDL**	**Cardiometabolic**	0.99931	0.98035	0.94681	0.99837	0.79800	0.72323	0.87379	0.51868	0.54810
**LDL**	**Cardiometabolic**	0.99934	0.96580	0.94192	0.99762	0.87185	0.83866	0.93087	0.58026	0.61421
**TC**	**Cardiometabolic**	0.99828	0.96481	0.94585	0.99644	0.85873	0.82793	0.88282	0.55328	0.58102
**CVD**	**Cardiometabolic**	0.96875	0.84621	0.89376	0.97184	0.78492	0.72526	0.85420	0.55300	0.58718
**Asthma**	**Allergies and autoimmune diseases**	0.98437	0.80848	0.83019	0.99243	0.70293	0.67279	0.82488	0.49850	0.52147
**Autoimmune_broad**	**Allergies and autoimmune diseases**	0.98243	0.86121	0.94386	0.99166	0.70707	0.68738	0.81360	0.47716	0.49501
**Autoimmune_certain**	**Allergies and autoimmune diseases**	0.97481	0.86865	0.94284	0.99364	0.79462	0.78258	0.78175	0.43352	0.44896
**Dermatologic**	**Allergies and autoimmune diseases**	0.95679	0.71144	0.68905	0.97947	0.69341	0.61992	0.78796	0.44955	0.46914
**Eczema**	**Allergies and autoimmune diseases**	0.98878	0.80788	0.83777	0.99228	0.66255	0.62723	0.83866	0.51729	0.54068
**Respiratory_ENT**	**Allergies and autoimmune diseases**	0.97941	0.80570	0.82230	0.99108	0.70611	0.66582	0.82348	0.49214	0.52040
**FEV1**	**Lung traits**	0.98986	0.91560	0.91623	0.98667	0.81804	0.76439	0.90881	0.61185	0.64439
**FVC**	**Lung traits**	0.96063	0.82182	0.87010	0.97020	0.80614	0.75616	0.89694	0.53461	0.56740
**PEF**	**Lung traits**	0.99865	0.95188	0.93782	0.99654	0.79732	0.76088	0.88197	0.56713	0.59684
**Average**		0.98191	0.87818	0.88120	0.98396	0.78102	0.73348	0.87415	0.55990	0.58792

Supplementary Table 3. Comparison of h^2 estimates for different traits in the UKB. Local heritability estimates from different methods are compared to those from BOLT-REML.

LD approx method	Bandwidth	Low-rank factors	Runtime
Band_only	50	0	5.943s
Band_only	100	0	6.250s
Band_only	200	0	5.007s
Band_only	300	0	4.983s
Band_only	400	0	5.388s
Band_only	500	0	5.345s
Band_only	1000	0	4.798s
LR_only	0	5	7.0m:8.579s
LR_only	0	50	6.0m:56.99s
LR_only	0	100	7.0m:18.57s
LR_only	0	500	6.0m:8.284s
LR_only	0	1000	8.0m:7.017s
LR_only	0	1500	8.0m:28.37s
LR_only	0	2000	9.0m:24.93s
LR_only	0	2500	9.0m:31.55s
Spike_LR	1	5	8.0m:31.67s
Spike_LR	1	50	6.0m:10.74s
Spike_LR	1	100	6.0m:55.27s
Spike_LR	1	500	7.0m:14.70s
Spike_LR	1	1000	7.0m:12.19s
Spike_LR	1	1500	9.0m:31.47s
Spike_LR	1	2000	16.0m:36.6s
Spike_LR	1	2500	16.0m:19.2s
Spike_PSD	1	5	1.0m:24.35s
Spike_PSD	1	50	2.0m:13.37s
Spike_PSD	1	100	2.0m:24.63s
Spike_PSD	1	500	5.0m:28.58s
Spike_PSD	1	1000	9.0m:21.92s
Spike_PSD	1	1500	16.0m:31.0s
Spike_PSD	1	2000	18.0m:42.3s
Spike_PSD	1	2500	29.0m:50.2s
Spike_PSD_hetero	1	5	2.0m:41.15s
Spike_PSD_hetero	1	50	3.0m:34.83s
Spike_PSD_hetero	1	100	3.0m:38.50s
Spike_PSD_hetero	1	500	3.0m:36.63s
Spike_PSD_hetero	1	1000	7.0m:24.90s
Spike_PSD_hetero	1	1500	20.0m:6.09s
Spike_PSD_hetero	1	2000	34.0m:54.2s
Spike_PSD_hetero	1	2500	55.0m:18.9s

Supplementary Table 4. Computational runtime of LD approximation from different decomposition methods. Existing methods are shown here. Details about the LD approximation methods are provided in Table 2.

LD approx method	Bandwidth	Low-rank factors	Runtime
Seq_Band_LR	100	300	10.0m:48.7s
Seq_Band_LR	100	400	9.0m:53.39s
Seq_Band_LR	100	500	7.0m:50.01s
Seq_Band_LR	200	300	10.0m:38.8s
Seq_Band_LR	200	400	7.0m:33.67s
Seq_Band_LR	200	500	6.0m:39.22s
Seq_Band_LR	300	300	7.0m:13.97s
Seq_Band_LR	300	400	8.0m:43.00s
Seq_Band_LR	300	500	6.0m:46.87s
Seq_Band_LR	400	200	8.0m:50.46s
Seq_Band_LR	400	300	8.0m:40.62s
Seq_Band_LR	400	400	8.0m:31.86s
Seq_Band_LR	400	500	6.0m:45.27s
Seq_Band_LR	500	50	6.0m:30.59s
Seq_Band_LR	500	100	11.0m:26.7s
Seq_Band_LR	500	500	6.0m:34.69s
Seq_Band_LR	500	1000	7.0m:4.890s
PSD_Band_LR	100	300	1.0h:18.0m:9.613s
PSD_Band_LR	100	400	1.0h:17.0m:13.19s
PSD_Band_LR	100	500	49.0m:44.796s
PSD_Band_LR	200	300	3.0h:18.0m:27.60s
PSD_Band_LR	200	400	1.0h:32.0m:57.22s
PSD_Band_LR	200	500	1.0h:18.0m:14.19s
PSD_Band_LR	300	300	5.0h:46.0m:5.179s
PSD_Band_LR	300	400	3.0h:23.0m:32.11s
PSD_Band_LR	300	500	2.0h:12.0m:16.52s
PSD_Band_LR	400	200	8.0h:16.0m:14.08s
PSD_Band_LR	400	300	8.0h:30.0m:38.97s
PSD_Band_LR	400	400	5.0h:8.0m:0.2597s
PSD_Band_LR	400	500	4.0h:22.0m:24.74s
PSD_Band_LR	500	50	2.0h:59.0m:29.66s
PSD_Band_LR	500	100	3.0h:43.0m:0.852s
PSD_Band_LR	500	500	3.0h:34.0m:3.144s
PSD_Band_LR	500	1000	3.0h:43.0m:15.26s
Joint_Band_LR	100	300	1.0h:22.0m:38.60s
Joint_Band_LR	100	400	2.0h:12.0m:57.36s
Joint_Band_LR	100	500	2.0h:20.0m:1.932s
Joint_Band_LR	200	300	1.0h:18.0m:2.648s
Joint_Band_LR	200	400	1.0h:16.0m:8.012s
Joint_Band_LR	200	500	1.0h:13.0m:59.21s
Joint_Band_LR	300	300	1.0h:10.0m:6.221s
Joint_Band_LR	300	400	1.0h:27.0m:7.220s
Joint_Band_LR	300	500	2.0h:44.0m:56.81s
Joint_Band_LR	400	200	57.0m:19.657s
Joint_Band_LR	400	300	55.0m:0.858s
Joint_Band_LR	400	400	1.0h:24.0m:44.12s
Joint_Band_LR	400	500	1.0h:24.0m:49.81s
Joint_Band_LR	500	50	1.0h:31.0m:28.27s
Joint_Band_LR	500	100	53.0m:9.754s
Joint_Band_LR	500	500	1.0h:12.0m:48.46s
Joint_Band_LR	500	1000	1.0h:30.0m:45.11s

Supplementary Table 5. Computational runtime of LD approximation from different decomposition methods. The Banded + LR methods are shown here. Details about the LD approximation methods are provided in Table 2.

Supplementary References

References

1. Yang, J. *et al.* Common snps explain a large proportion of the heritability for human height. *Nat. genetics* **42**, 565–569 (2010).
2. Loh, P.-R. *et al.* Contrasting genetic architectures of schizophrenia and other complex diseases using fast variance-components analysis. *Nat. genetics* **47**, 1385 (2015).
3. Speed, D., Hemani, G., Johnson, M. R. & Balding, D. J. Improved heritability estimation from genome-wide snps. *The Am. J. Hum. Genet.* **91**, 1011–1021 (2012).
4. Evans, L. M. *et al.* Comparison of methods that use whole genome data to estimate the heritability and genetic architecture of complex traits. *Nat. genetics* **50**, 737–745 (2018).
5. Gazal, S., Marquez-Luna, C., Finucane, H. K. & Price, A. L. Reconciling s-ldsc and ldak functional enrichment estimates. *Nat. genetics* **51**, 1202–1204 (2019).
6. Haseman, J. & Elston, R. The investigation of linkage between a quantitative trait and a marker locus. *Behav. genetics* **2**, 3–19 (1972).
7. Chen, G.-B. Estimating heritability of complex traits from genome-wide association studies using ibs-based haseman–elston regression. *Front. genetics* **5**, 107 (2014).
8. Sham, P. C. & Purcell, S. Equivalence between haseman-elston and variance-components linkage analyses for sib pairs. *The Am. J. Hum. Genet.* **68**, 1527–1532 (2001).
9. Wu, Y. & Sankararaman, S. A scalable estimator of snp heritability for biobank-scale data. *Bioinformatics* **34**, i187–i194 (2018).
10. Guan, Y. & Stephens, M. Bayesian variable selection regression for genome-wide association studies and other large-scale problems. (2011).
11. Zhou, X., Carbonetto, P. & Stephens, M. Polygenic modeling with bayesian sparse linear mixed models. *PLoS genetics* **9**, e1003264 (2013).
12. de Los Campos, G., Hickey, J. M., Pong-Wong, R., Daetwyler, H. D. & Calus, M. P. Whole-genome regression and prediction methods applied to plant and animal breeding. *Genetics* **193**, 327–345 (2013).
13. Habier, D., Fernando, R. L., Kizilkaya, K. & Garrick, D. J. Extension of the bayesian alphabet for genomic selection. *BMC bioinformatics* **12**, 1–12 (2011).
14. Bulik-Sullivan, B. K. *et al.* Ld score regression distinguishes confounding from polygenicity in genome-wide association studies. *Nat. genetics* **47**, 291–295 (2015).

15. Lee, J. J., McGue, M., Iacono, W. G. & Chow, C. C. The accuracy of ld score regression as an estimator of confounding and genetic correlations in genome-wide association studies. *Genet. epidemiology* **42**, 783–795 (2018).

16. Holmes, J. B., Speed, D. & Balding, D. J. Summary statistic analyses can mistake confounding bias for heritability. *Genet. Epidemiol.* **43**, 930–940 (2019).

17. Speed, D. & Balding, D. J. Sumher better estimates the snp heritability of complex traits from summary statistics. *Nat. genetics* **51**, 277–284 (2019).

18. Gazal, S. *et al.* Linkage disequilibrium–dependent architecture of human complex traits shows action of negative selection. *Nat. genetics* **49**, 1421–1427 (2017).

19. Zhou, X. A unified framework for variance component estimation with summary statistics in genome-wide association studies. *The annals applied statistics* **11**, 2027 (2017).

20. Hou, K. *et al.* Accurate estimation of snp-heritability from biobank-scale data irrespective of genetic architecture. *Nat. genetics* **51**, 1244–1251 (2019).

21. Shi, H., Kichaev, G. & Pasaniuc, B. Contrasting the genetic architecture of 30 complex traits from summary association data. *The Am. J. Hum. Genet.* **99**, 139–153 (2016).

22. Zhou, W. *et al.* Efficiently controlling for case-control imbalance and sample relatedness in large-scale genetic association studies. *Nat. genetics* **50**, 1335–1341 (2018).

23. Wen, X. & Stephens, M. Using linear predictors to impute allele frequencies from summary or pooled genotype data. *The annals applied statistics* **4**, 1158 (2010).

24. Zhu, X. & Stephens, M. Bayesian large-scale multiple regression with summary statistics from genome-wide association studies. *The annals applied statistics* **11**, 1561 (2017).

25. Ning, Z., Pawitan, Y. & Shen, X. High-definition likelihood inference of genetic correlations across human complex traits. *Nat. genetics* **52**, 859–864 (2020).

26. Zhang, Y. *et al.* Comparison of methods for estimating genetic correlation between complex traits using gwas summary statistics. *Briefings bioinformatics* **22**, bbaa442 (2021).

27. Harville, D. A. Maximum likelihood approaches to variance component estimation and to related problems. *J. Am. statistical association* **72**, 320–338 (1977).

28. Henderson, C. R. Maximum likelihood estimation of variance components. *Unpubl. manuscript. Ithaca, NY: Dep. Animal Sci. Cornell Univ.* **14850** (1973).

29. Henderson, C. Estimation of variances in animal model and reduced animal model for single traits and single records. *J. Dairy Sci.* **69**, 1394–1402 (1986).

30. Anderson, T. W. Statistical inference for covariance matrices with linear structure. *Multivar. Analysis II* 55–66 (1969).

31. Patterson, H. D. & Thompson, R. Recovery of inter-block information when block sizes are unequal. *Biometrika* **58**, 545–554 (1971).

32. Patterson, H. Maximum likelihood estimation of components of variance. In *Proceeding Eight International Biometric Conference, 1975* (Biometric Soc., 1975).

33. Searle, S. R., Casella, G. & McCulloch, C. E. *Variance components*, vol. 391 (John Wiley & Sons, 2009).

34. Dempster, A. P., Laird, N. M. & Rubin, D. B. Maximum likelihood from incomplete data via the em algorithm. *J. Royal Stat. Soc. Ser. B (Methodological)* **39**, 1–22 (1977).

35. Wu, C. J. On the convergence properties of the em algorithm. *The Annals statistics* 95–103 (1983).

36. Halko, N., Martinsson, P.-G. & Tropp, J. A. Finding structure with randomness: Stochastic algorithms for constructing approximate matrix decompositions, 2009. *URL http://arxiv. org/abs/0909.4061. oai: arXiv. org* **909** (2009).

37. Schoech, A. P. *et al.* Quantification of frequency-dependent genetic architectures in 25 uk biobank traits reveals action of negative selection. *Nat. communications* **10**, 790 (2019).
