# Supplementary material for: Accurate and efficient estimation of local heritability using summary statistics and the linkage disequilibrium matrix
Source: Nat Commun. 2023 Dec 2;14:7954. doi: 10.1038/s41467-023-43565-9 (PMC10692177; doi:10.1038/s41467-023-43565-9)
Supplement: Supplementary file 3 — Reporting Summary [file 41467_2023_43565_MOESM3_ESM.pdf]

nature portfolio

Corresponding author(s): Xihong Lin

Last updated by author(s): 10/13/2023

Reporting Summary

Nature Portfolio wishes to improve the reproducibility of the work that we publish. This form provides structure for consistency and transparency in reporting. For further information on Nature Portfolio policies, see our Editorial Policies and the Editorial Policy Checklist.

Statistics

For all statistical analyses, confirm that the following items are present in the figure legend, table legend, main text, or Methods section.

n/a	Confirmed	
☐	☒	The exact sample size (*n*) for each experimental group/condition, given as a discrete number and unit of measurement
☒	☐	A statement on whether measurements were taken from distinct samples or whether the same sample was measured repeatedly
☐	☒	The statistical test(s) used AND whether they are one- or two-sided<br>*Only common tests should be described solely by name; describe more complex techniques in the Methods section.*
☐	☒	A description of all covariates tested
☐	☒	A description of any assumptions or corrections, such as tests of normality and adjustment for multiple comparisons
☐	☒	A full description of the statistical parameters including central tendency (e.g. means) or other basic estimates (e.g. regression coefficient) AND variation (e.g. standard deviation) or associated estimates of uncertainty (e.g. confidence intervals)
☒	☐	For null hypothesis testing, the test statistic (e.g. *F*, *t*, *r*) with confidence intervals, effect sizes, degrees of freedom and *P* value noted<br>*Give P values as exact values whenever suitable.*
☒	☐	For Bayesian analysis, information on the choice of priors and Markov chain Monte Carlo settings
☒	☐	For hierarchical and complex designs, identification of the appropriate level for tests and full reporting of outcomes
☐	☒	Estimates of effect sizes (e.g. Cohen's *d*, Pearson's *r*), indicating how they were calculated

Our web collection on statistics for biologists contains articles on many of the points above.

Software and code

Policy information about availability of computer code

Data collection	We did not collect data for this study. We analyzed raw genotype-phenotype data from UKBB (application 52008).
Data analysis	Our method (HEELS) has been implemented as an open-source Python package, available at: https://github.com/huilisabrina/HEELS<br>Access to the UK Biobank resource is available via application (http://www.ukbiobank.ac.uk/).<br>Both HEELS and the following software packages were used in simulation studies and real data analyses:<br>-- GREML or the GCTA tool (https://yanglab.westlake.edu.cn/software/gcta/#Download)<br>-- LD Score regression (https://github.com/bulik/ldsc)<br>-- GRE (https://github.com/bogdanlab/h2-GRE)<br>-- HESS (https://github.com/huwenboshi/hess)<br>-- BOLT-LMM v2.3.4 is available at https://data.broadinstitute.org/alkesgroup/BOLT-LMM/<br>-- We also used Python (3.6.3) to perform statistical analyses and used R (3.6.3) for data visualization.

For manuscripts utilizing custom algorithms or software that are central to the research but not yet described in published literature, software must be made available to editors and reviewers. We strongly encourage code deposition in a community repository (e.g. GitHub). See the Nature Portfolio guidelines for submitting code & software for further information.

Data

Policy information about availability of data

All manuscripts must include a data availability statement. This statement should provide the following information, where applicable:

- Accession codes, unique identifiers, or web links for publicly available datasets
- A description of any restrictions on data availability
- For clinical datasets or third party data, please ensure that the statement adheres to our policy

TThis work uses the genotypic and phenotypic data from the UK Biobank study (\href{https://www.ukbiobank.ac.uk}{https://www.ukbiobank.ac.uk}). Our access to the individual-level data was approved under application number 52008.

Research involving human participants, their data, or biological material

Policy information about studies with human participants or human data. See also policy information about sex, gender (identity/presentation), and sexual orientation and race, ethnicity and racism.

Reporting on sex and gender	Sex was used as a covariate to control for in the analysis model. Sex was determined based on the agreement of self-reporting and genetically determined (presence of Y chromosome) information provided by the UK biobank.
Reporting on race, ethnicity, or other socially relevant groupings	The analyses were conducted using the individuals who self-identified as White British in the UK Biobank, so that the results will not be confounded by population stratifications.
Population characteristics	Participants in the UK Biobank data (http://www.ukbiobank.ac.uk/), de-identified publicly available data. Since we used the subset of N=332K unrelated White British-ancestry individuals in the UK Biobank, the population characteristics of our samples (in terms of age, disease diagnosis and genotypic information) are similar to the UK Biobank superset of white British.
Recruitment	UK Biobank recruited 500,000 people aged between 40-69 years in 2006-2010 from across the UK. More details can be found at http://www.ukbiobank.ac.uk/.
Ethics oversight	UK Biobank data were obtained under the application number 52008. The UK Biobank EGF (https://www.ukbiobank.ac.uk/the-ethics-and-governance-council/) provides the required ethics oversight.

Note that full information on the approval of the study protocol must also be provided in the manuscript.

Field-specific reporting

Please select the one below that is the best fit for your research. If you are not sure, read the appropriate sections before making your selection.

☒ Life sciences ☐ Behavioural & social sciences ☐ Ecological, evolutionary & environmental sciences

For a reference copy of the document with all sections, see nature.com/documents/nr-reporting-summary-flat.pdf

Life sciences study design

All studies must disclose on these points even when the disclosure is negative.

Sample size	We analyzed publicly available UK Biobank data of independent white British samples (N=332,430). We also used various subsets of this sample for various simulation studies in order to accommodate the existing methods that are not scalable to biobank-sized dataset. As this is a method paper, we did not perform any sample size calculation. As illustrated in data analysis results, the very large sample size of the UK Biobank provide heritability estimates with small standard errors.
Data exclusions	Our heritability estimation method requires independent subjects in homogeneous population. Following common practices in heritability estimation, we excluded non White British subjects and related subjects in the analysis of the UK Biobank data, and also excluded the sex chromosome from all of our analyses and analyzed only autosomes.
Replication	No replication dataset was analyzed. This is a methods paper. We illustrated the proposed method and compared its performance with the existing methods by analysis of the large UK biobank data.
Randomization	The UK Biobank is an observational study. To account for potentially confounding factors such as population structures, age and sex, we estimated heritability by controlling for covariates in all of our statistical analyses, including genetic ancestry principal components (top 40 PCs, which is standard in this type of analyses), sex, age and age squared.
Blinding	We used de-identified coded data, and hence were blinded.

Reporting for specific materials, systems and methods

We require information from authors about some types of materials, experimental systems and methods used in many studies. Here, indicate whether each material, system or method listed is relevant to your study. If you are not sure if a list item applies to your research, read the appropriate section before selecting a response.

Materials & experimental systems

n/a	Involved in the study
☒	☐ Antibodies
☒	☐ Eukaryotic cell lines
☒	☐ Palaeontology and archaeology
☒	☐ Animals and other organisms
☒	☐ Clinical data
☒	☐ Dual use research of concern
☒	☐ Plants

Methods

n/a	Involved in the study
☒	☐ ChIP-seq
☒	☐ Flow cytometry
☒	☐ MRI-based neuroimaging
